# Author Correction: Drug-dependent growth curve reshaping reveals mechanisms of antifungal resistance in *Saccharomyces cerevisiae*

**DOI:** 10.1038/s42003-022-03648-7

**Published:** 2022-07-12

**Authors:** Lesia Guinn, Evan Lo, Gábor Balázsi

**Affiliations:** 1 The Louis and Beatrice Laufer Center for Physical and Quantitative Biology, Stony Brook University, Stony Brook, NY 11794 USA; 2 Department of Biomedical Engineering, Stony Brook University, Stony Brook, NY 11794 USA

**Keywords:** Antifungal agents, Antimicrobial resistance, Differential equations, Fungal genetics, Data processing

Correction to: *Communications Biology* 10.1038/s42003-022-03228-9, published online 31 March 2022.

The original version of this Article contained an error in Fig. 2f, in which the incorrect axis label and values were included for the second graph (“Growth Curve Phasal Breakdown”). The correct version of Fig. 2f is:
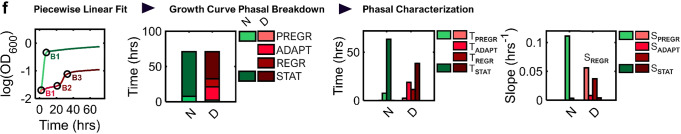


which replaces the previous incorrect version:
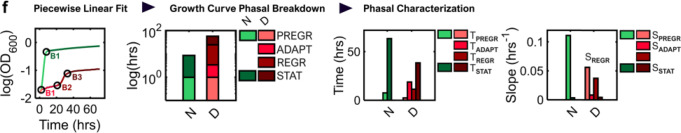


This has now been corrected in both the PDF and HTML versions of the Article.

