# Supplementary material for: Drug-dependent growth curve reshaping reveals mechanisms of antifungal resistance in Saccharomyces cerevisiae
Source: Commun Biol. 2022 Mar 31;5:292. doi: 10.1038/s42003-022-03228-9 (PMC8971432; doi:10.1038/s42003-022-03228-9)
Supplement: Supplementary file 2 — Supplementary Information [file 42003_2022_3228_MOESM2_ESM.pdf]

SUPPLEMENTARY INFORMATION:

Drug-Dependent Growth Curve Reshaping Reveals Mechanisms of Antifungal Resistance in *Saccharomyces cerevisiae* Lesia Guinn[1,2], Evan Lo[1], Gábor Balázsi[1,2]*

[1]Laufer Center for Physical and Quantitative Biology, Stony Brook University, Stony Brook, NY, 11794, USA

[2]Department of Biomedical Engineering, Stony Brook University, Stony Brook, NY, 11794, USA

Keywords:

multicellularity, yeast, antifungal drugs, drug resistance, growth curve analysis, mathematical modeling

***

SUPPLEMENTARY NOTES

Supplementary Note 1. Estimating cell counts from absorbance (OD_{600}) measurements 2
Supplementary Note 2. Mathematical models of cell growth without stress 4
Supplementary Note 2.1 Modeling growth dynamics - basics 4
Supplementary Note 2.2 Comparison with experimental data supports sugar-limited Allee growth . 4
Supplementary Note 2.3. Parameters of growth dynamics without stressors 5
Supplementary Note 3. Mathematical models of cell growth with stress 6
Supplementary Note 4. Response of TBR1 EvoTop cells to stressors 7
7. Supplementary References 37

Supplementary Note 1. Estimating cell counts from absorbance (OD_{600}) measurements

We compared OD600 absorbance measurements and cell counts at $n_D = 8$ twofold serial dilutions. Overall dilution factors $D = 2^{n_D}$ increased from 1-fold to 128-fold for each of the 4 cell types. Data analysis revealed biases both in cell counts and OD600 measurements.

First, serial dilutions must cause a hyperbolic dependence of cell counts *N* on dilution factor *D* as shown in Equation (1). Any deviations from the hyperbolic dependence must be due to experimental artefacts.

$$N = \frac{N_0}{D} = \frac{N_0}{2^{n_D}} \quad (1)$$

Taking the logarithm gives the expected linear dependence of log cell counts on log dilution factors for *D*=1,2,4,8,…,128 as in Equation (2):

$$ln(N) = ln(N_0) - ln(D) = ln(N_0) - n_D\, ln(2) \quad (2)$$

Therefore, linear fits to $ln(N)$ versus $ln(D)$ must have a slope = -1.

The corresponding plots and linear fits are in **Supplementary Figure 5a**. All slopes are greater than -1, indicating an instrumental/experimental bias. The most likely causes of cell count underestimates at high cell densities are cells with overlapping cross-sections and the cells pushed outside of the focal plane. Cell counts at low cell densities are expected to be most accurate and will be used to eliminate biases at high cell densities. Besides the unexpectedly gradual slope, there are systematic deviations from linearity (**Supplementary Figure 5a**). Our first goal was to restore a linear $y = ln(N)$ versus $x = ln(D)$ dependence and a slope -1.

Using *cftool* in MATLAB, we observed that a double-exponential function $f = a\, e^{bx} + c\, e^{dx}$ fits the $y = ln(N)$ versus $x = ln(D)$ data well. The fit parameters are shown in **Supplementary Table 2**.

Next, we obtained error estimates $\varepsilon_i = y_i - f_i$ by subtracting these double-exponential estimates f_i from the data y_i. Then we added these error estimates back to the corrected cell count function in Equation (3). This correction restored the expected linear $\tilde{y} = ln(\widetilde{N})$ versus $x = ln(D)$ dependence with the slope of -1. Interestingly, the corrected log(TBR1 cell counts) were lower by an average of 1.484274490243554 than for the other 3 strains. We corrected this deviation expected from TBR1's clumpy nature, which causes the cell counting program to miss cells clumped in 3D structures, outside of the focal plane, or cells individually discernable within clumps (**Supplementary Figure 5a**).

$$\tilde{y}_i = ln(\widetilde{N}_i) = \varepsilon_i + ln(N_8) + (i - 8)\, ln(2) \quad (3)$$

Next, we sought a relationship that would estimate the corrected cell count $\tilde{y}_i = ln(\tilde{N}_i)$ for every OD600 absorbance value $z_i = ln(a_i)$. Using MATLAB's *cftool*, we observed that the function shown in Equation (4) provided reliable corrected cell count estimates. The fit parameters are in **Supplementary Table 3**, and the estimates are indicated by "+" symbols in the **Supplementary Figure 5b**. Subsequently we applied these corrections to estimate cell counts from OD measurements for each strain (**Supplementary Figure 5b**).

$$\hat{y}_i = \alpha(z_i + 2.5)^\beta + \gamma(z_i + 4)^\delta \quad (4)$$

Supplementary Note 2. Mathematical models of cell growth without stress

Supplementary Note 2.1 Modeling growth dynamics - basics

Simple models of cell population growth relate the number of cells$N(t)$ and sugar concentration $S(t)$ starting from initial conditions $N(0) = N_0$ and $S(0) = S_0$ through equations assuming sugar conversion into new cells: $-\frac{dS}{dt} = \frac{dN}{dt} = U(S, N)$. Possible choices for $U(S, N)$ are the Monod model: $U(S, N) = \frac{cSN}{M+S}$; the logistic model: $U(S, N) = cSN$; the Allee effect: $U(S, N) = cSN(N + C)$; or uptake-limited exponential growth: $U(S, N) = cN$.

This model conveys the idea that cells convert sugar into biomass. When this conversion is sugar-limited, the final cell count should depend linearly on the initial sugar amount (**Supplementary Figure 6**). This is also true for frequency-dependent growth models, such as growth with Allee effect that yeast is known to show in glucose[1], which fit our data best (Equation 5):

$$-\frac{dS}{dt} = \frac{dN}{dt} = cSN(N + C) \tag{5}$$

Applying the constraint $S(t) + N(t) = S_0 + N_0$ results in the Equation 6:

$$\frac{dN}{dt} = cN(S_0 + N_0 - N)(N + C) \tag{6}$$

The parameters in Equation 6 are the following: $S_0 + N_0 = K$ is the carrying capacity;N_0 is the initial population size; c is the sugar uptake rate, and C is the Allee parameter.

Supplementary Note 2.2 Comparison with experimental data supports sugar-limited Allee growth

For total sugar-limited growth, the difference between the final and initial cell counts (or cell count increment) should be proportional to the initial sugar level: $N_f - N_0 = \alpha S_0 + \beta$. We tested this possibility. Linear fits to final cell count estimates from experimental OD data was strong and gave for TBR1 wild type, α=[3.3809 ± 0.10454]x10^8/sugar unit and β=[0.6975 ± 0.14619]x10^8, while for TBR1Δa, α=[3.4054 ± 0.1181]x10^8/sugar unit and β=[1.0542 ± 0.05542]x10^8.

Therefore, the sugar unit of 1% glucose (3.342662 x10^{19} molecules/mL) is converted into an average $\langle\alpha\rangle$ of 3.3932x10^8 cells/mL during growth, so creating a new cell requires 9.85106 x10^{10} glucose molecules, or 1.6358 x10^{-13} moles of glucose.

Importantly, the intercept of the cell increment versus sugar exceeds zero. This means that even in 0% sugar, some cell growth is possible. This is expected if cells resuspended from a sugar-containing medium have internalized some sugar and have accumulated internal resources that allow some cells to still divide.

Indeed, dividing the intercept with the initial cell counts indicates that the population size could still increase by 0.87585 $x10^8$ cells starting from $1.4681x10^8$ cells/mL upon resuspension in YPD medium without any sugar. In other terms, each yeast cell carries internal resources equivalent to generating 0.5965874 cells, or equivalent to ~$5.877x10^{10}$ external molecules of glucose/cell in these experiments. We validated this prediction experimentally (top row of panels in the previous figure).

Supplementary Note 2.3. Parameters of growth dynamics without stressors

Armed with an understanding of sugar utilization, we can now model growth curves in sugar. Curve fitting with two free parameters indicated that growth models with an Allee effect captured the data best as shown in Equation 5. The best fit TBR1 parameters are listed in the **Supplementary Table 4**.

Supplementary Note 3. Mathematical models of cell growth with stress

To model the effect of stress, we modify the previous sugar-only growth equation. The variables represent concentrations. When the external drug E enters the cell, it becomes internal drug D, which causes toxicity T to accumulate with a rate proportional to D. Toxicity inhibits cell growth according to a Hill function $\frac{q}{q+T}$, which equally slows down the dilution rate of intracellular drug D. The cell senses the toxicity and tries to remove it with a saturating enzymatic rate of $p\frac{T}{r+T}$. We also consider that toxicity causes sugar uptake-dependent or independent cell death at rates bT and kNT, respectively.

$$\frac{dN}{dt} = c\frac{q}{q+T}NS(N+C) - kNT \quad (7)$$

or for fluconazole:

$$\frac{dN}{dt} = \left(c\frac{q}{q+T} - bT\right)NS(N+C) - kNT \quad (8)$$

$$\frac{dS}{dt} = -cNS(N+C)\frac{q}{q+T} \quad (9)$$

$$\frac{dD}{dt} = f(E-D) - cDS(N+C)\frac{q}{q+T} - dD \quad (10)$$

$$\frac{dT}{dt} = aD - p\frac{T}{r+T} \quad (11)$$

$$\frac{dE}{dt} = -dE - f(E-D)\frac{Nv}{V-Nv} \quad (12)$$

We used a scaling factor $\frac{Nv}{V-Nv}$ in Equation (12) to account for the difference in the volume v of cell interiors and V of the growth medium. Thus, a small amount of drug can increase the internal drug concentration D substantially while it may not cause a large concentration loss in the E concentration in the external medium. We assumed that the drug spontaneously degraded with rate d both inside and outside the cells.

For each increasing drug concentration series, we first fit the growth curves in 0 stress, to extract the two sugar utilization parameters (sugar-cell affinity c and Allee effect parameter C) as well as the effective (intracellular + extracellular) sugar amount in cell equivalents using $N_f - N_0 = \alpha S_0 + \beta$. Next, we kept all sugar utilization parameters constant and introduced the stress effects in Equations (7)-(12) into the model. We initiate the fits with wide constraints for all parameters, which we gradually tighten up based on the quality of individual fits (**Supplementary Table 5**). We also enforce that the spontaneous drug decay rate stays identical in all strains and all drug concentrations, once determined in TBR1.

Supplementary Note 4. Response of TBR1 EvoTop cells to stressors

Whereas unicellularity due to *AMN1* deletion in the TBR1 background generally decreased drug resistance compared to TBR1, the antifungal drug response of experimentally evolved unicellular TBR1 EvoTop cells[2] has not yet been tested. We therefore investigated the response of the TBR1 EvoTop strain (**Figure 1B, D-E**, **Supplementary Figure 15-16**) to the same stressors.

The stressors caused similar growth curve reshaping in TBR1 EvoTop as in TBR1 and TBR1Δa, but at much higher (e.g., up to 10-fold higher H_2O_2) concentrations than the other strains. In TBR1Δa, 0.02% was the highest H_2O_2 concentration resulting in regrowth, whereas TBR1 EvoTop cells survived a maximum of 0.6% H_2O_2 exposure (**Supplementary Figure 15b, 16a**), higher concentrations causing total growth arrest. The adaptation phase duration grew sharper than in the TBR1Δa as a function of peroxide concentration. The final absorbance at the last survived dose, however, increased more for TBR1 EvoTop than in TBR1Δa (STAT OD_{600}, **Supplementary Figure 16a**). The extension of adaptation phase with higher stress was consistent among all three strains.

To explore how TBR1 EvoTop responds to specific antifungals, we exposed TBR1 EvoTop to the same concentrations of AmB and CASP as we used for TBR1 and TBR1Δa treatment (**Supplementary Figure 15c-d,, 16b-c**). We once again confirmed the drug-specific dynamic, which in the case of AmB was the emergence of two new phases – an additional adaptation and a shortened regrowth phase (**Supplementary Figure 15c, 16b**). The drop in carrying capacity was also noticeable starting at the 0.8 µg/ml dose (compared to 0.6 µg/ml for the unicellular knock-out strain). CASP-induced curve smoothing was observed in this strain as well as in the parental TBR1, characterized by decreasing slopes pregrowth and adaptation at higher drug concentrations (**Supplementary Figure 16c**).

Name	Oligonucleotide sequence	Template
**LR1.2.1**	GACTGGGTTGGAAGGCAAGAGGAACTAACAAAAACGTTCAAAAAGTTT	TBR1 Genomic DNA
**LR1.2.2**	GGTATTCTGGGCCTCCATGTCACTTTGATCTGTTTTTAAAGACAAATCT	TBR1 Genomic DNA
**LR1.2.3**	TGATTACGCCAAGCTTGCATGAACACTATGAGTGTATCTTTTGGAGAAT	TBR1 Genomic DNA
**LR1.2.4**	TGAATGCTGGTCGCTATACTGAACTCATTTCCAAATCTGGCTGTTTTAG	TBR1 Genomic DNA
**LR1.2.5**	AGATTTGTCTTTAAAAACAGATCAAAGTGACATGGAGGCCCAGAATACC	pFA6-KanMX2
**LR1.2.6**	CTAAAACAGCCAGATTTGGAAATGAGTTCAGTATAGCGACCAGCATTCA	pFA6-KanMX2
**LR1.2.7**	CAGGCCAGCCATTACGCTCGTCAT	pFA6-KanMX2
**LR1.2.8**	ATCCCCGGCAAAACAGCATTCCAG	pFA6-KanMX2
**LR1.2.9**	CATGCAAGCTTGGCGTAATCA	pBYO11
**LR1.2.10**	CTCTTGCCTTCCAACCCAGTC	pBYO11
**LR1.2.11**	GTTATGGCTTAACGTAACAAGGCCTTT	*AMN1*
**LR1.2.12**	TGACACTCCATTCTGGAAAGCCTTTCT	*AMN1*

Supplementary Table 1. Primers used in this study. Primers shown here were used to perform the HiFi assembly (LR1.2.1-2 for left *AMN1* homology arm, LR1.2.3-4 for right *AMN1* homology arm, LR1.2.5-6 for *KanMX*) and sequencing (LR1.2.7-12) of the *AMN1* knock-out plasmid and of the *AMN1* genomic locus in *S. cerevisiae* TBR1 (LR1.2.11-12).

	a	*b*	*c*	*d*
TBR1	13.71 (-272.4, 299.9)	-0.1132 (-2.102, 1.876)	5.317 (-281.1, 291.8)	0.04337 (-3.485, 3.572)
TBR1Δa	18.47 (10.84, 26.1)	-0.07636 (-0.1969, 0.0442)	0.8763 (-6.886, 8.638)	0.2739 (-0.7706, 1.318)
BY4742	2.12 (-5.331, 9.57)	-0.5061 (-2.025, 1.013)	17.33 (9.795, 24.87)	-0.02075 (-0.08686, 0.04537)
BY4742Δa	0.5387 (-1.341, 2.419)	-0.9205 (-4.904, 3.063)	19.21 (17.3, 21.12)	-0.03831 (-0.0593, -0.01734)

Supplementary Table 2. Parameter estimates from fitting the double-exponential function $f = a\,e^{bx} + c\,e^{dx}$ to the serial dilution cell count data in **Supplementary Figure 5**.

	α	β	γ	δ
TBR1	13.44 (-32.29, 59.16)	0.2996 (-1.566, 2.165)	17.01 (8.425, 25.6)	-1.769 (-16.55, 13.01)
TBR1Δa	18.2 (17.88, 18.51)	0.09755 (0.07606, 0.119)	1.011e-05 (-0.0002221, 0.0002424)	7.783 (-7.611, 23.18)
BY4742	-14.59 (-18.2, -10.97)	1.43 (1.236, 1.624)	6.346 (4.726, 7.966)	1.771 (1.37, 2.171)
BY4742Δa	17.26 (16.76, 17.77)	0.09956 (0.06473, 0.1344)	0.001068 (-0.009153, 0.01129)	4.826 (-1.064, 10.72)

Supplementary Table 3. Parameter estimates from fitting the function $\hat{y}_i = \alpha(z_i + 2.5)^{\beta} + \gamma(z_i + 4)^{\delta}$ to the log(OD600 values z_i).

Sugar (glucose)	0%=0 TBR1 cells	0.5%= 169.66 x10^6 cells	1%=339.32 x10^6 cells	2%=678.64 x10^6 cells
c (/cell^2/h)	1.51 x10^{-4}	0. 14 x10^{-4}	0.045 x10^{-4}	0.016 x10^{-4}
C (cells)	-26.39 x10^6	-129.43 x10^6	-94.56 x10^6	-65.70 x10^6
S_T (cells) = N_f	74.15 x10^6	377.19 x10^6	541.23 x10^6	873.96 x10^6

Supplementary Table 4. The parameters that produce the best fit to the TBR1 0-stress curves at various glucose concentrations. The columns represent conditions with various sugar content (0, 0.5, 1, and 2% glucose).

Symbol	Meaning	Parameter Bound	H_2O_2		AmB		CASP		FLC	
			TBR1	TBR1Δa	TBR1	TBR1Δa	TBR1	TBR1Δa	TBR1	TBR1Δa
q	Threshold to inhibit growth (concentration)	*Lower*	*3E-6*	*0.0075*	*0.003*	*0.001*	*E-4*	*E-6*	*0.01*	*1.0*
		Upper	*0.003*	*0.075*	*0.01*	*0.004*	*0.001*	*0.001*	*0.1*	*2.5*
f	Drug influx rate (h^{-1})	*Lower*	*0.1*	*1.25*	*0.01*	*0.03*	*0.005*	*0.025*	*0.005*	*0.025*
		Upper	*0.5*	*2.5*	*0.02*	*0.06*	*0.01*	*0.05*	*0.02*	*0.05*
a	Toxicity accumulation rate (h^{-1})	*Lower*	*1.5*	*4.0*	*0.4*	*1.0*	*3.0*	*1.5*	*15.0*	*15.0*
		Upper	*4.0*	*15.0*	*0.8*	*2.0*	*5.5*	*8.0*	*35.0*	*35.0*
p	Maximum detox rate (h^{-1})	*Lower*	*0.5*	*0.015*	*0.3*	*0.05*	*2.0*	*0.25*	*5.0*	*1.0*
		Upper	*0.8*	*0.03*	*0.5*	*0.3*	*2.5*	*1.0*	*10.0*	*10.0*
r	Threshold to trigger detox (concentration)	*Lower*	*1.0*	*2.5*	*1.5*	*0.7*	*0.3*	*0.1*	*1.0*	*5.0*
		Upper	*5.0*	*3.5*	*3.0*	*1.7*	*0.8*	*5.0*	*30.0*	*10.0*
d	Drug decay rate (h^{-1})	*Lower*	*1.0*	*1.0*	*0.1*	*0.1*	*0.01*	*0.01*	*0.2*	*0.2*
		Upper	*1.1*	*1.1*	*0.2*	*0.2*	*0.015*	*0.015*	*0.25*	*0.25*
k	Sugar-independent killing rate (h^{-1})	*Lower*	*0.001*	*0.3*	*0.1*	*0.1*	*1.0*	*0.5*	*3E-6*	*3E-6*
		Upper	*0.5*	*1.0*	*0.25*	*0.25*	*2.0*	*1.5*	*1E-5*	*0.001*
b	Sugar-dependent killing rate (h^{-1})	*Lower*	–	–	–	–	–	–	*4E-6*	*3E-4*
		Upper	–	–	–	–	–	–	*8E-6*	*0.001*

Supplementary Table 5. Parameter constraints used for TBR1 and TBR1Δa growth curve modelling. The parameters are estimated from the model using the initial sugar amount, cell count, and Allee effect from the prior control (0 stress, no-drug) experimental growth curve fit. "Concentration" units divided by parameter a match the units for each drug, except for H_2O_2 are 10-fold higher (10% instead of 1%) and for FLC are 1000-fold higher (mg/ml instead of µg/ml).

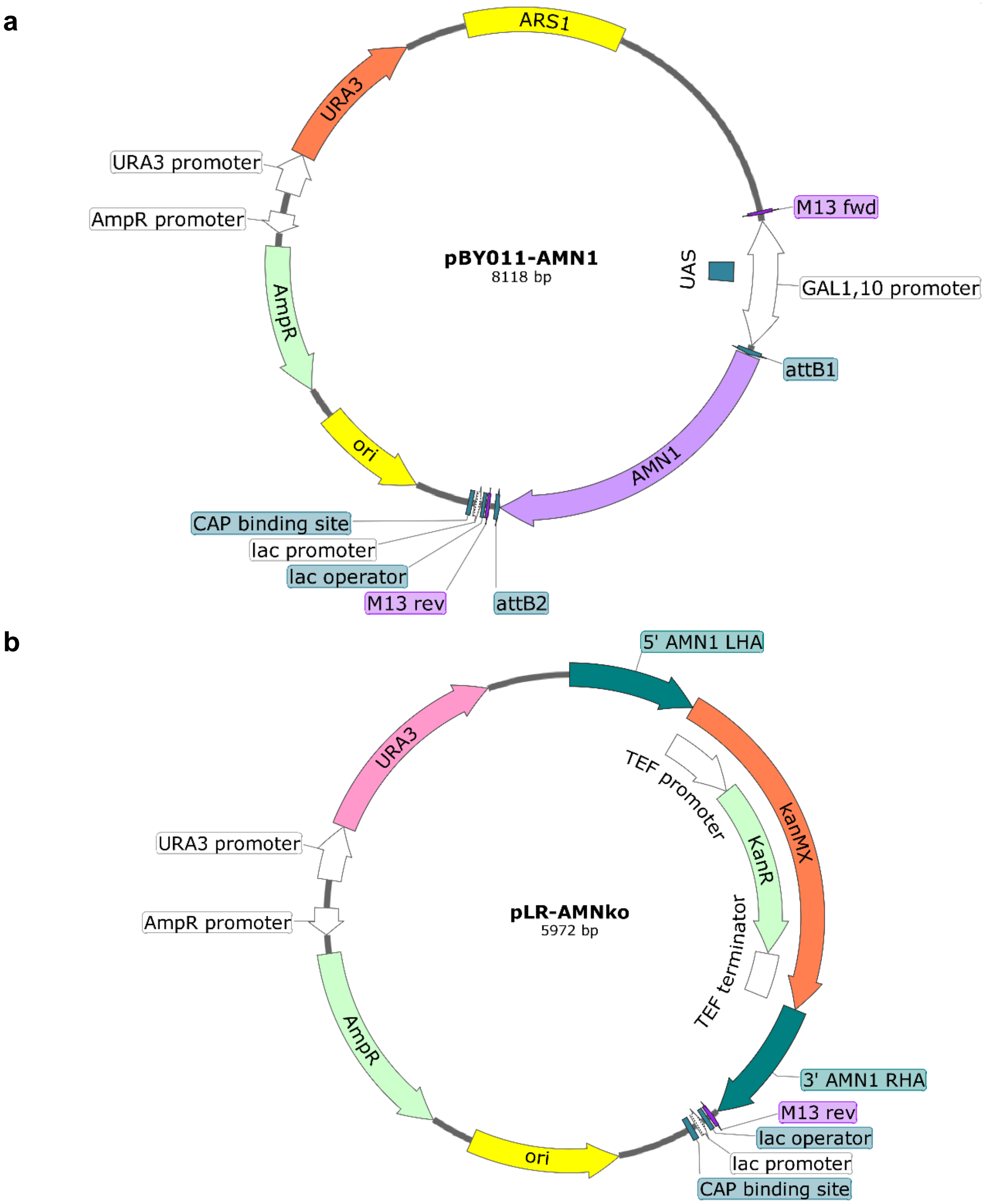

Supplementary Figure 1. *AMN1* template plasmid and *AMN1* knock-out plasmid. Schematic illustrations of the plasmids used/built in this study. **a)** *AMN1* mother vector, pBY011-*AMN1* (Harvard Plasmid ID # ScCD00097520). **b)** *AMN1* knock-out vector, pLR-AMNko.

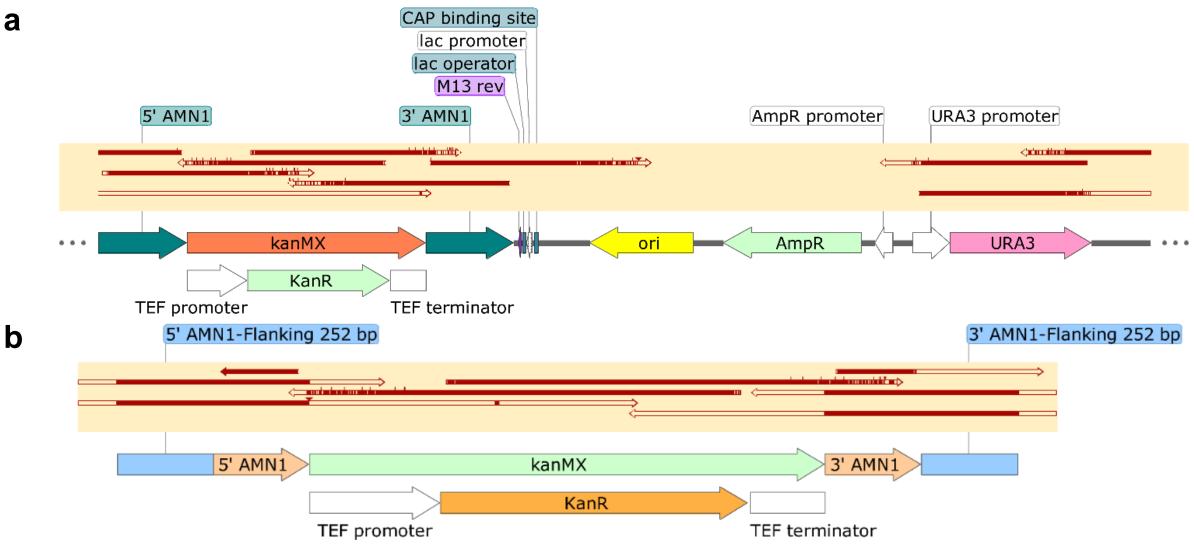

Supplementary Figure 2. *AMN1* knock-out sequencing. a) *AMN1* knock-out plasmid (pLR-AMNko) sequencing summary schematic. **b)** Knock-out genomic integration. The schematic of TBR1 genomic DNA sequencing in the *AMN1* locus.

155

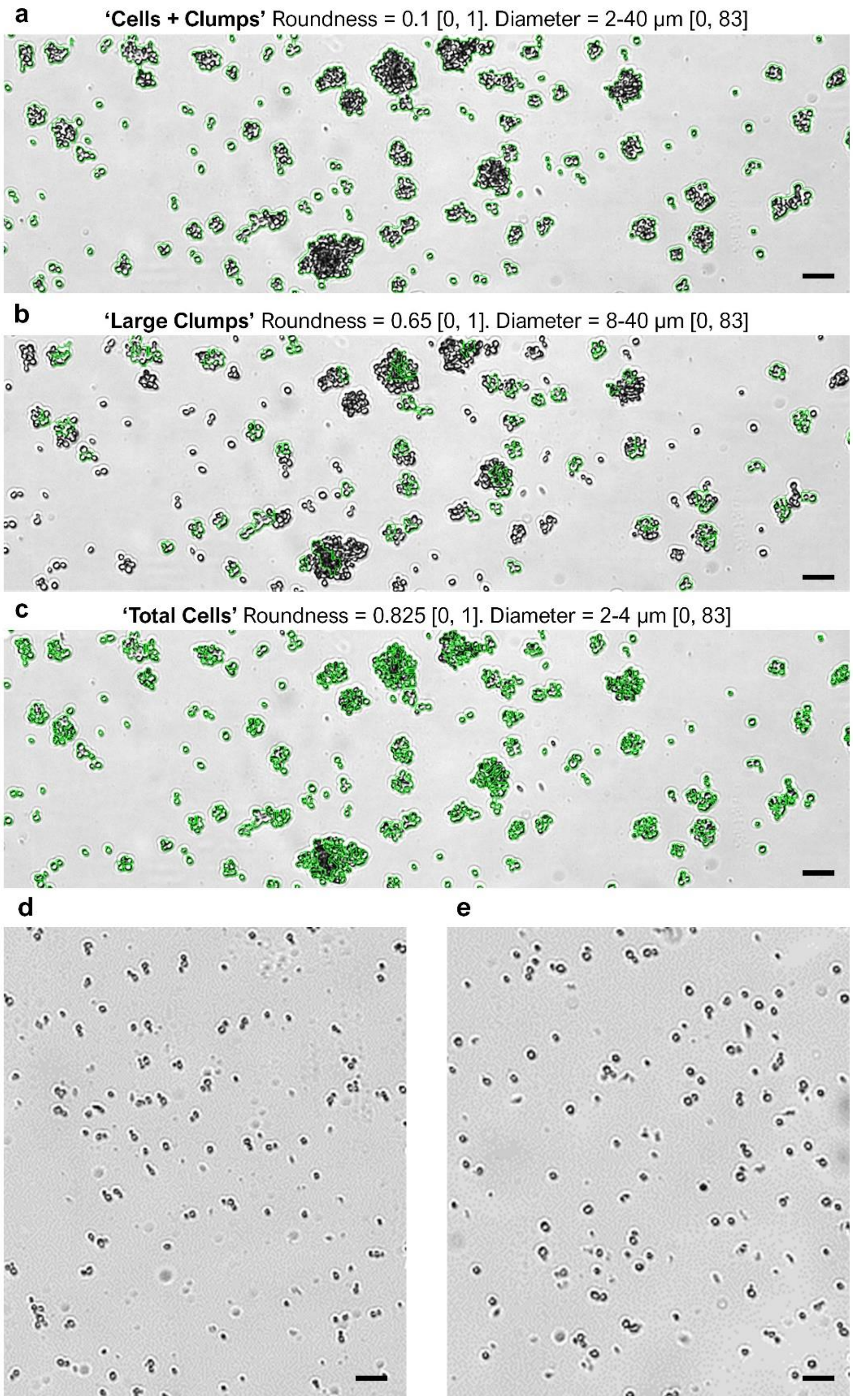

Supplementary Figure 3. Nexcelom Image Segmentation for single cell/clump identification. Developed in Cellometer® Vision CBA Image Cytometer (Nexcelom Bioscience LLC.), custom Image Segmentation Modes detected: **a)** both single cells and clumps, **b)** clumps only, and **c)** free single cells and single cells within clumps. Brightfield 10x microscope image of BY4742 **(d)** and BY4742Δa **(e)** laboratory strains grown in YPD. Scale bar = 10 µm.

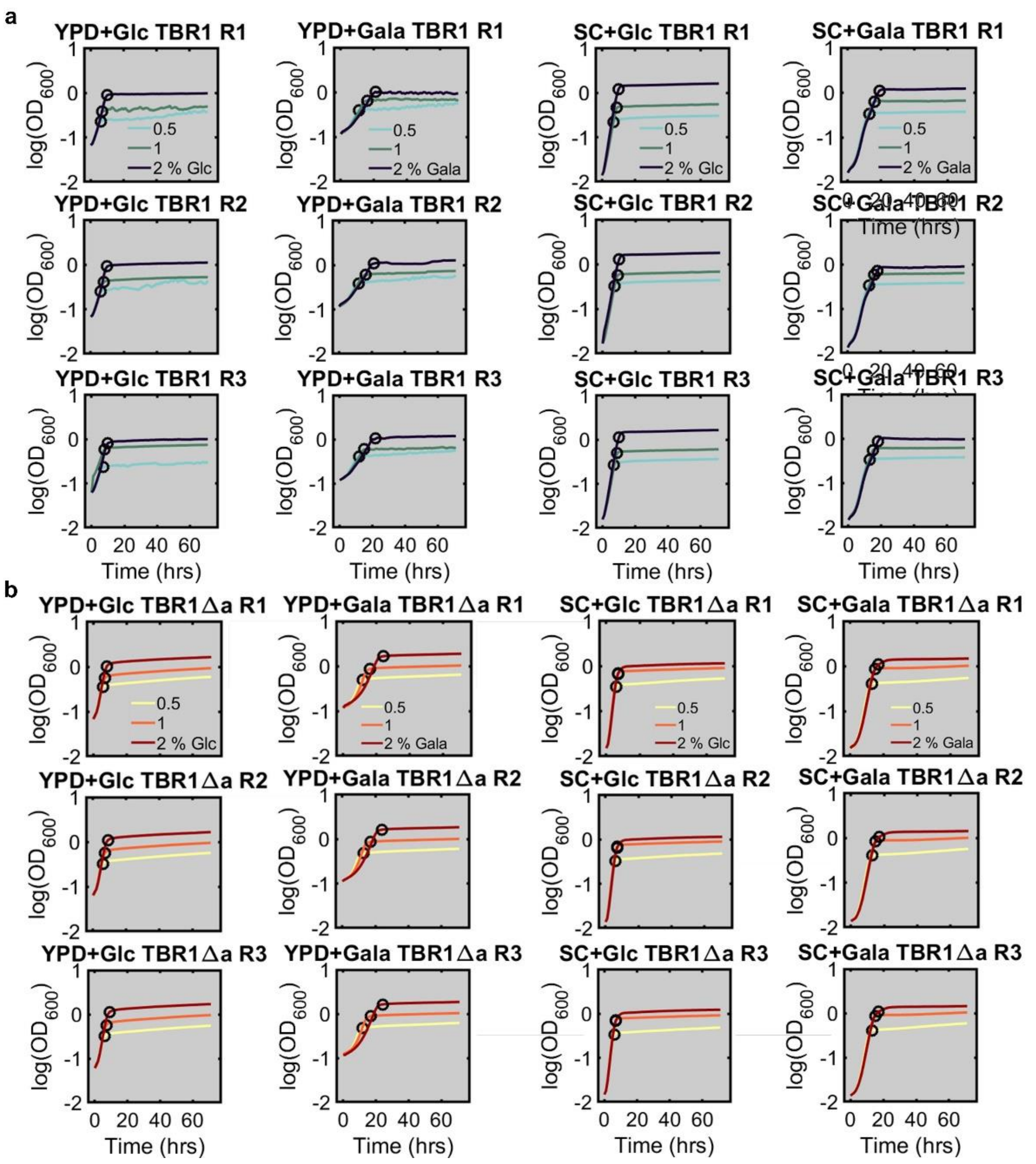

Supplementary Figure 4. Growth curves in various media without drugs. a) TBR1 and **b)** TBR1Δa growth curves (shown as means and confidence interval) in YPD and SC media with varying glucose and galactose content (0.5, 1, and 2%).

a

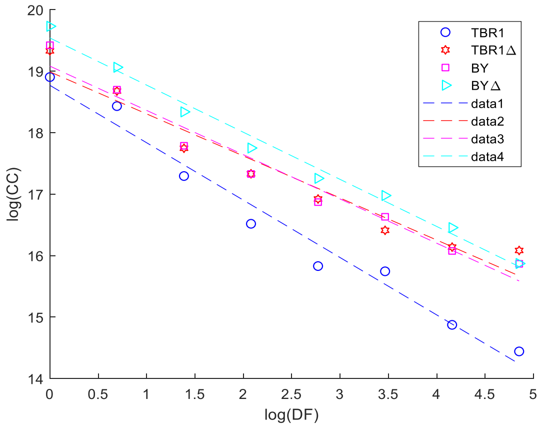

**Original**	**Slope**	**Intercept**	**RSS**
TBR1	-0.9335	18.7688	0.4517
TBR1Δa	-0.6839	18.9881	0.5283
BY4742	-0.7201	19.0812	0.4094
BY4742Δa	-0.7664	19.5388	0.1439

b

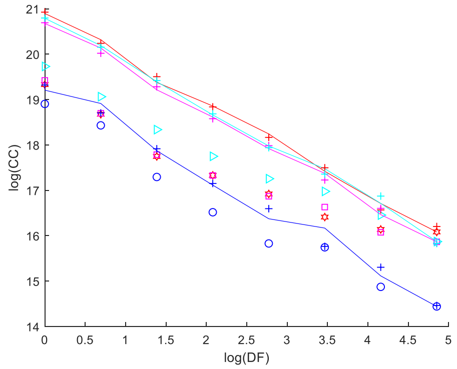

**Corrected**	**Slope**	**Intercept**	**RSS**
TBR1	-0.999571	19.325492	0.245931
TBR1Δa	-0.999852	20.913551	0.046640
BY4742	-1.000015	20.708716	0.05215
BY4742Δa	-1.000319	20.810601	0.043844

Supplementary Figure 5. Linear fits to the cell count data. a) Initial linear fits to the original log(cell counts) serial dilution data for the 4 yeast strains. The table on the right shows the linear fit parameters from MATLAB's *polyfit*. **b)** Corrected linear fits to the original log(cell counts) serial dilution data for the 4 yeast strains. Original (open symbols), corrected (lines), and OD-estimated log(cell counts) versus dilution factor for the 4 yeast strains. Cell count estimates from OD values indicated by "+" symbols are close to the corrected cell counts. The table shows linear fit parameters to the corrected data.

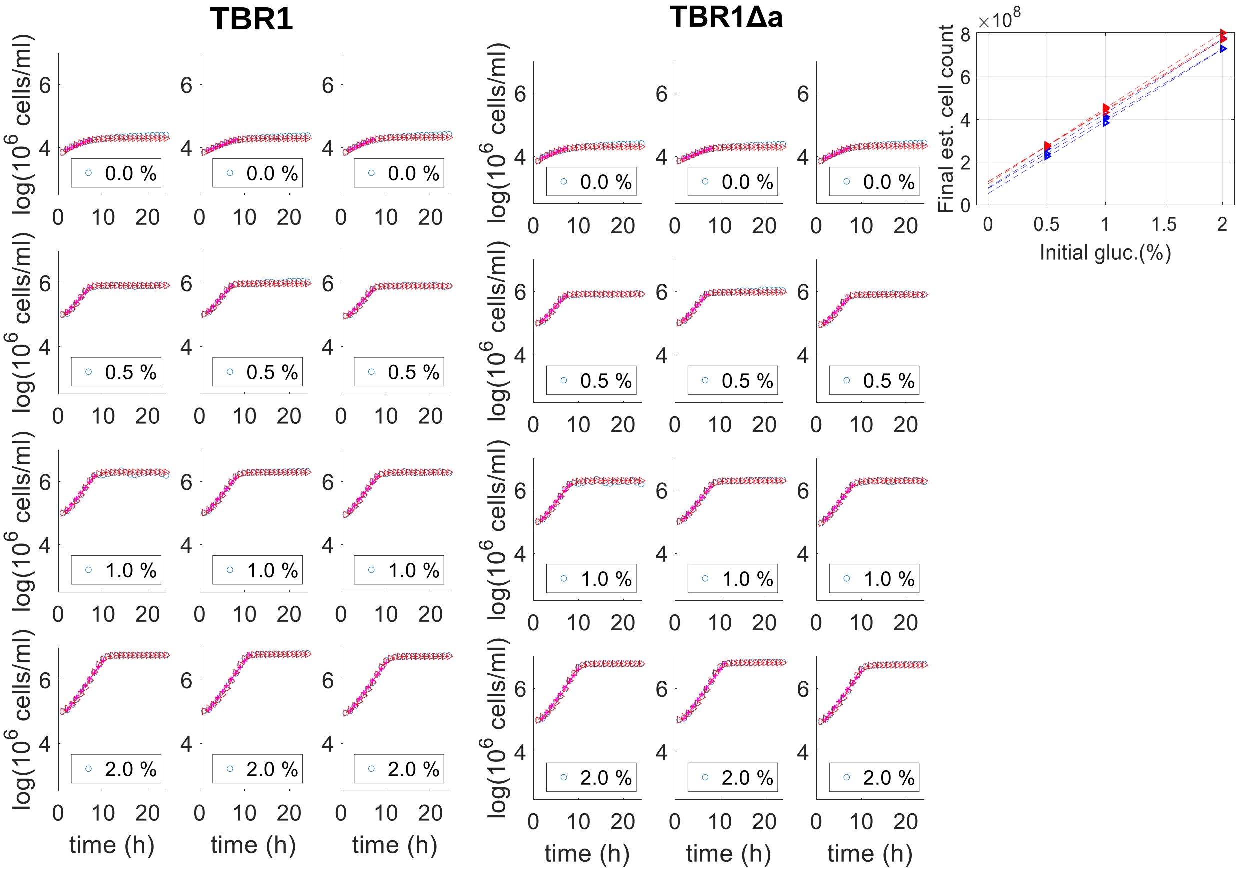

Supplementary Figure 6. Growth curves using cell count estimates depend on sugar content. Growth curves without stress in YPD medium for TBR1 and TBR1Δa indicate that the final cell count increment increases linearly with sugar concentrations, allowing the estimation of sugar to make a new cell. Blue circles are data points, red triangles are fits. Interestingly, the top row of panels indicates that cells carry some internal sugar and can grow when resuspended in YPD medium with 0% sugar.

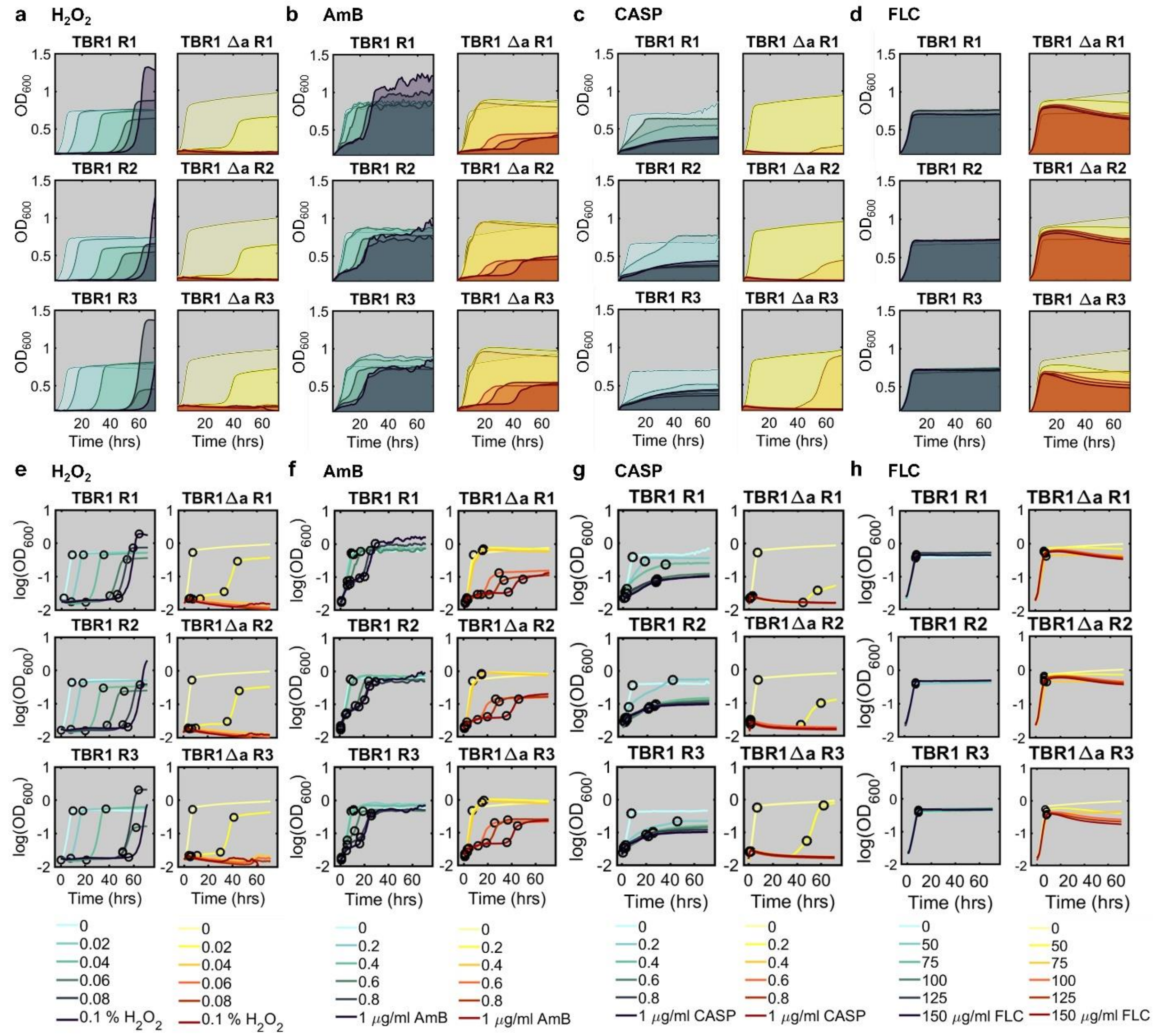

Supplementary Figure 7. Stress-dependent growth curve reshaping for TBR1 and TBR1Δa. (A-D) The area under each curve (indicating cumulative lifespan) upon exposure to **a)** hydrogen peroxide, H_2O_2, **b)** amphotericin B, AmB, **c)** caspofungin, CASP, and **D)** fluconazole, FLC, in increasing concentrations. **(e-h)** Semilogarithmic growth curves of TBR1 (blue) and TBR1Δa (yellow) strains analyzed with piecewise linear fitting in YPD containing **e)** hydrogen peroxide, H_2O_2, **f)** amphotericin B, AmB, **g)** caspofungin, CASP, and **h)** fluconazole, FLC, in sublethal/fungistatic concentrations listed at the bottom of each panel. Black circles indicate the breakpoints defined by the piecewise linear fitting.

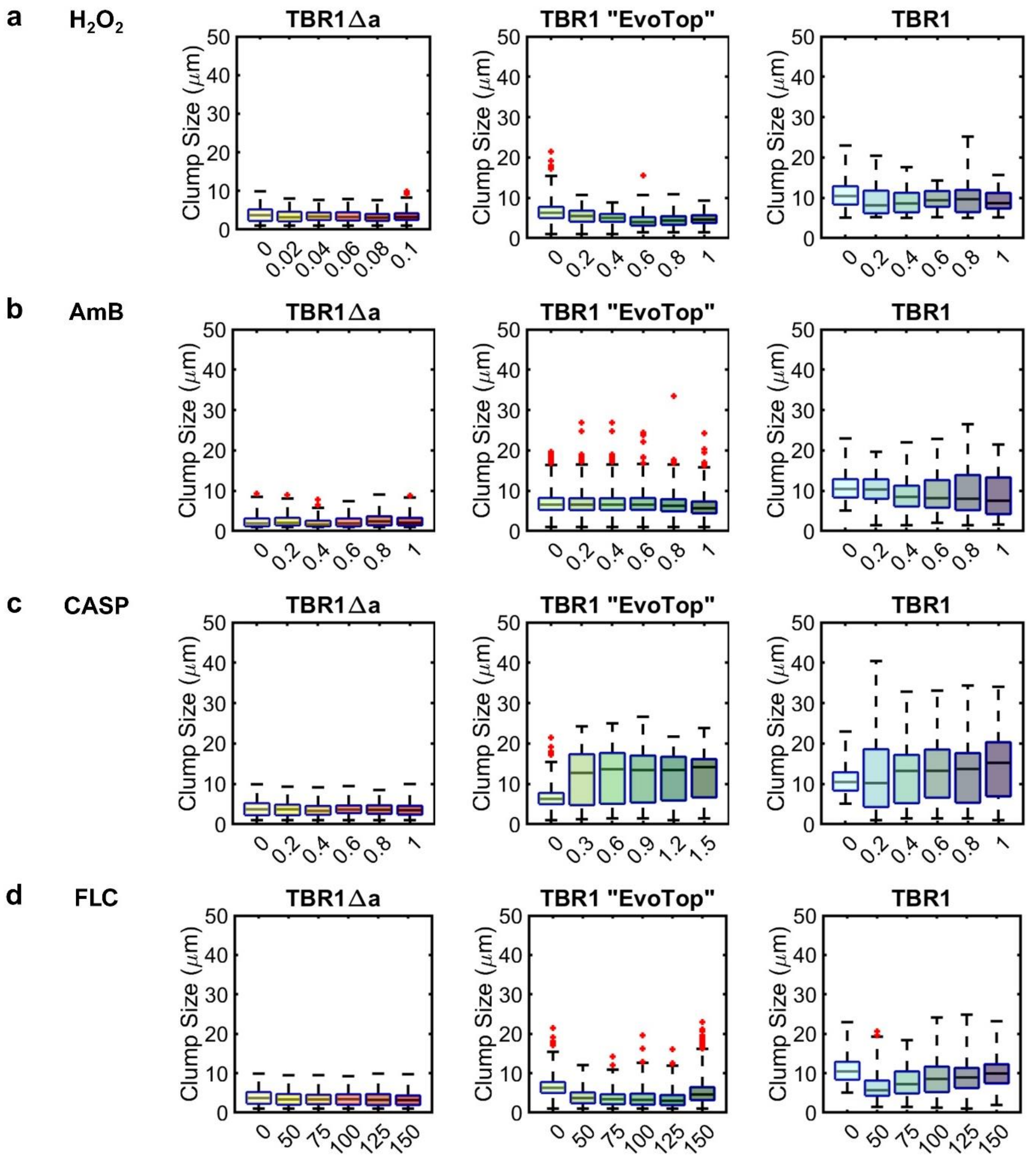

Supplementary Figure 8. Studying drug effects on clump/cell sizes. Clump (cell) sizes of TBR1, TBR1 EvoTop, and TBR1Δa upon **a)** H_2O_2, **b)** AmB, **c)** CASP, and **d)** FLC exposure shown as boxplots with whiskers extending to ±3 × IQR, a median and standard deviation error bars representing clump size from three sampled populations (n=4128). Single red dots representing the outliers.

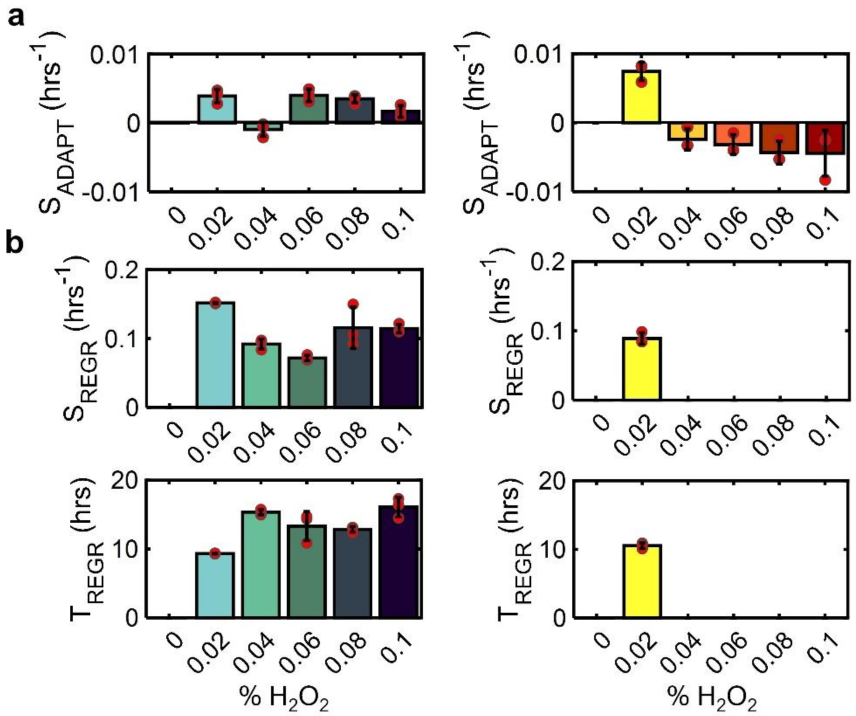

Supplementary Figure 9. Hydrogen peroxide (H_2O_2) effects on the most relevant growth phase parameters. a) The slope of the adaptation phase (T_{ADAPT}) of TBR1 (shades of blue) and TBR1Δa (shades of red) in various H_2O_2 concentrations. **b)** The slope and duration of the regrowth phase (S_{REGR}, T_{REGR}) in various H_2O_2 concentrations. Red circles represent individual data points. Error bars represent means and standard deviations calculated from three biological replicates. Complementary data is shown in **Figure 3C-D**.

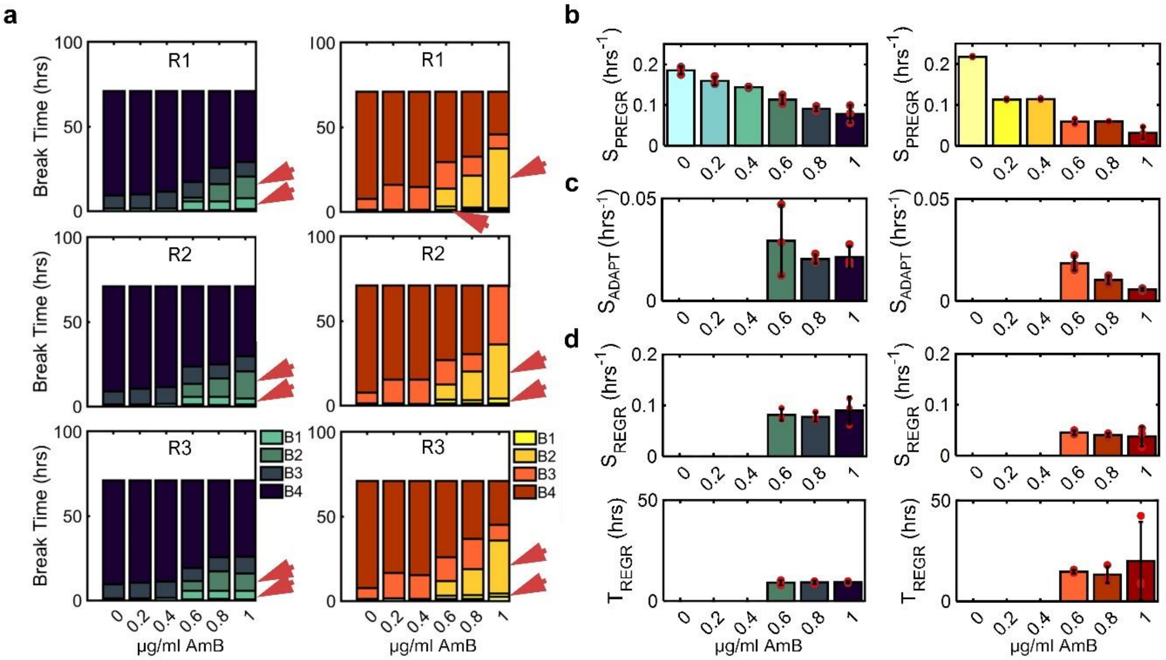

Supplementary Figure 10. Amphotericin B (AmB) effects on the most relevant growth phase parameters. a) Growth phase break time points (B1-4) defining the segments in TBR1 (shades of blue) and TBR1Δa (shades of red) growth curves defined by the piecewise linear fitting. The red arrows point at an additional (adaptation) growth phase produced by the 0.6, 0.8, and 1 µg/ml AmB concentrations. **b)** The slope of the pregrowth phase (S_{PREGR}) in various AmB concentrations. **c)** The slope of the adaptation phase (S_{ADAPT}) in various AmB concentrations. **d)** The slope and duration of the regrowth phase (S_{REGR}, T_{REGR}) in various AmB concentrations. Red circles represent individual data points. Error bars represent means and standard deviations calculated from three biological replicates. Complementary data is shown in **Figure 3G-I**.

176

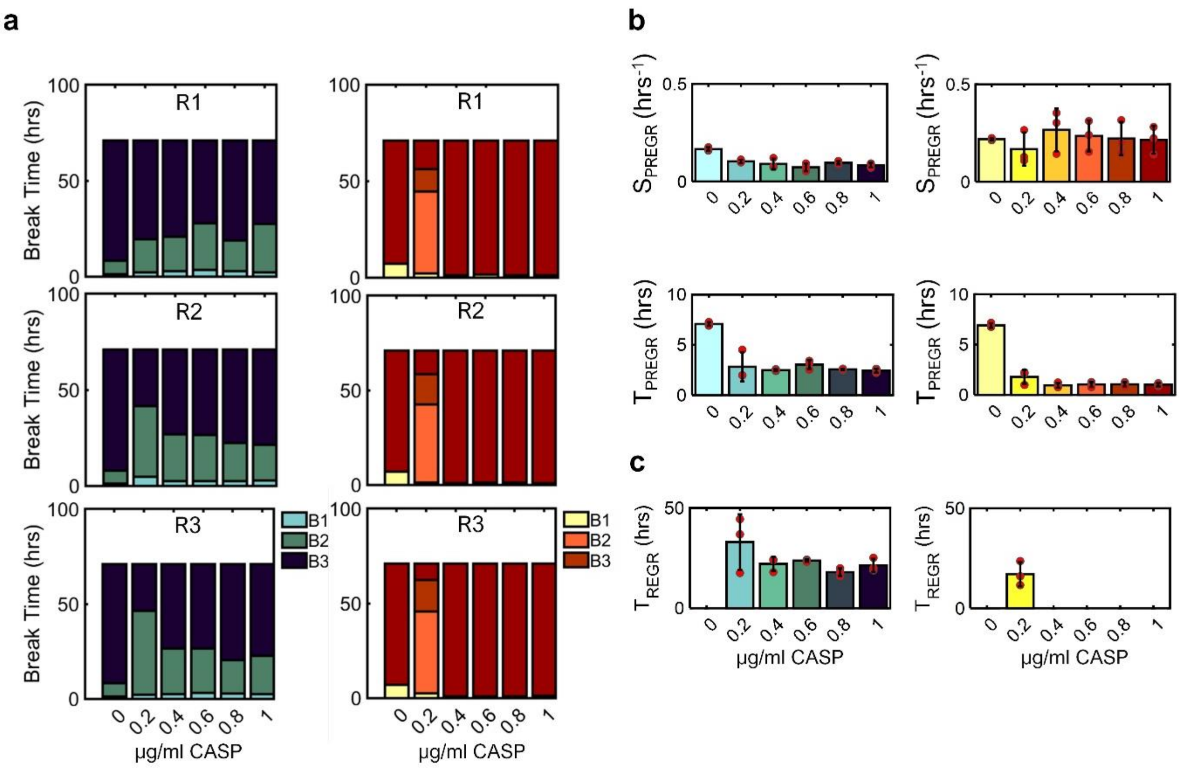

Supplementary Figure 11. Caspofungin (CASP) effects on the most relevant growth phase parameters. **a)** Growth phase break time points (B1-3) defining the segments in TBR1 (shades of blue) and TBR1Δa (shades of red) growth curves defined by the piecewise linear fitting. **b)** The slope and duration of the pregrowth phase (S_{PREGR}, T_{PREGR}) in various CASP concentrations. **c)** The duration of the regrowth phase (T_{REGR}) in various CASP concentrations. Red circles represent individual data points. Error bars represent means and standard deviations calculated from three biological replicates. Complementary data is shown in **Figure 4C-E**.

177

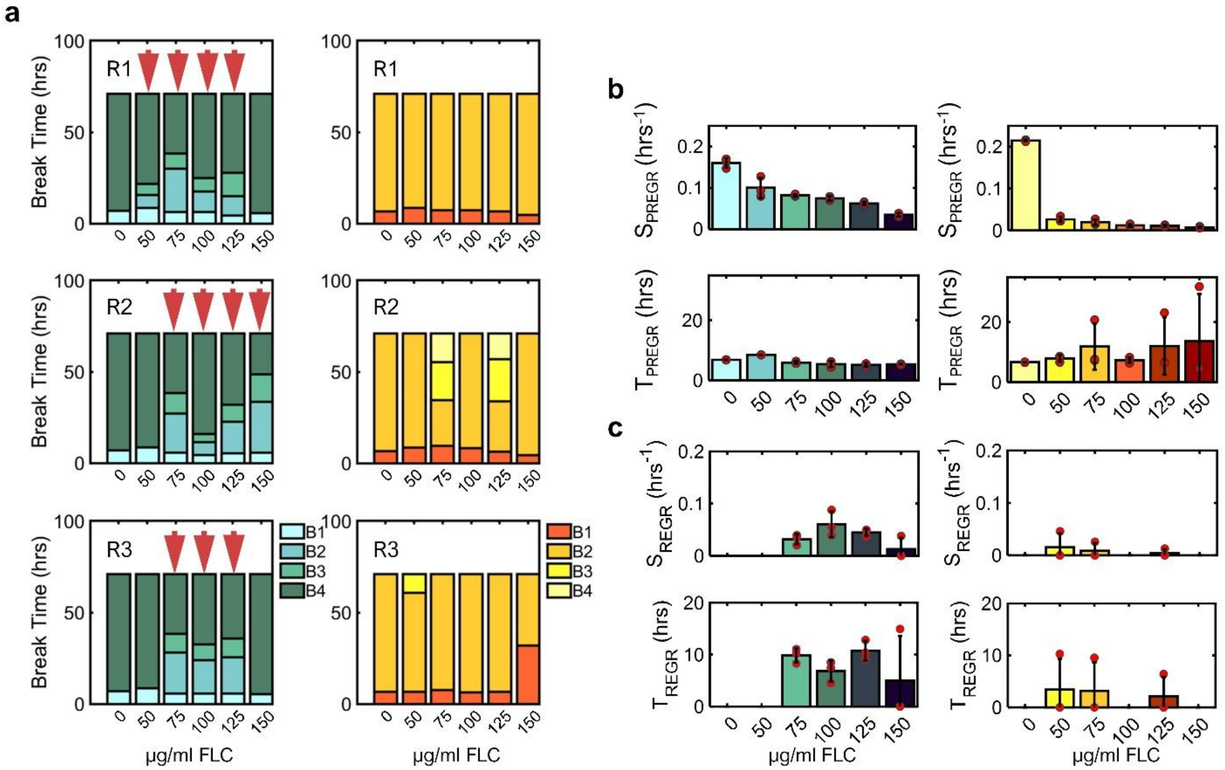

Supplementary Figure 12. Fluconazole (FLC) effects on the most relevant growth phase parameters. a) Growth phase break time points (B1-4) defining the segments in TBR1 (shades of green) and TBR1Δa (shades of yellow) growth curves after resuspension, defined by the piecewise linear fitting. The red arrows point at the drug concentrations that produced two additional growth phases. **b)** The slope and duration of the pregrowth phase (S_{PREGR}, T_{PREGR}) after resuspension of cells in the fresh drug-containing media after 10 hours of incubation. **c)** The slope and duration of the regrowth phase (S_{REGR}, T_{REGR}) after resuspension of cells in the fresh drug-containing media after 10 hours of incubation. Red circles represent individual data points. Error bars represent means and standard deviations calculated from three biological replicates. Complementary data is shown in **Figure 4H-J**.

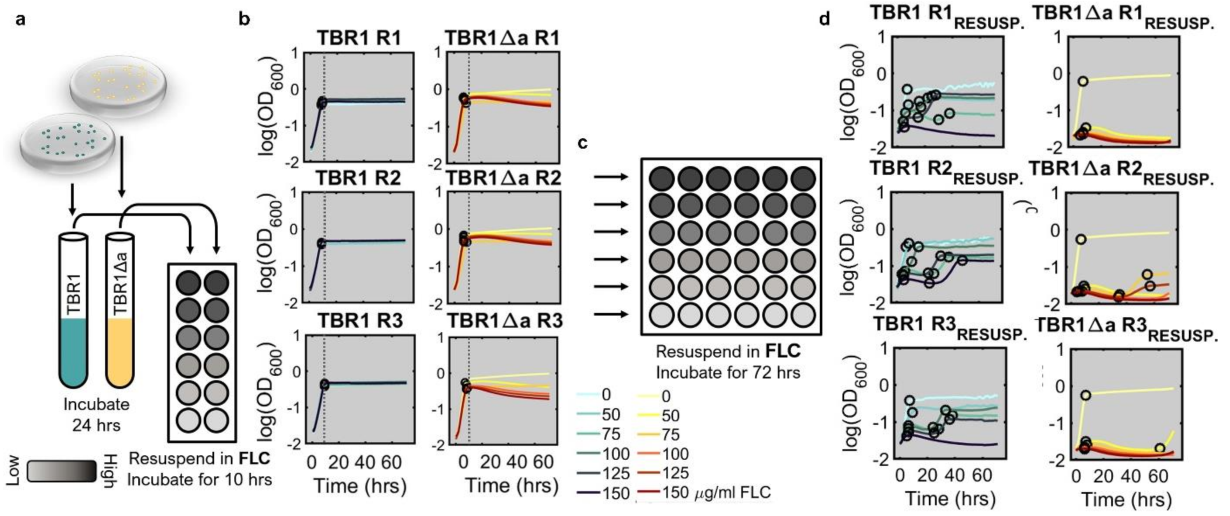

Supplementary Figure 13. Experimental FLC resuspension workflow. a) Illustration of the FLC exposure experimental design. B) TBR1 and TBR1Δa growth kinetics upon various FLC concentrations shown as log(OD_{600}) over 72 hours without resuspension. Dotted line marks 10 hours, when the cells were washed from the old media and transferred in the fresh FLC-containing media. Black circles indicate the breakpoints defined by the piecewise linear fitting. **c)** After 10 hours of initial incubation, some cells were transferred in the fresh solutions of correspondent drug doses and incubated for another 72 hours. **d)** TBR1 and TBR1Δa growth kinetics upon various FLC concentrations shown as log(OD_{600}) over 72 hours upon single drug exposure. Black circles indicate the breakpoints defined by the piecewise linear fitting.

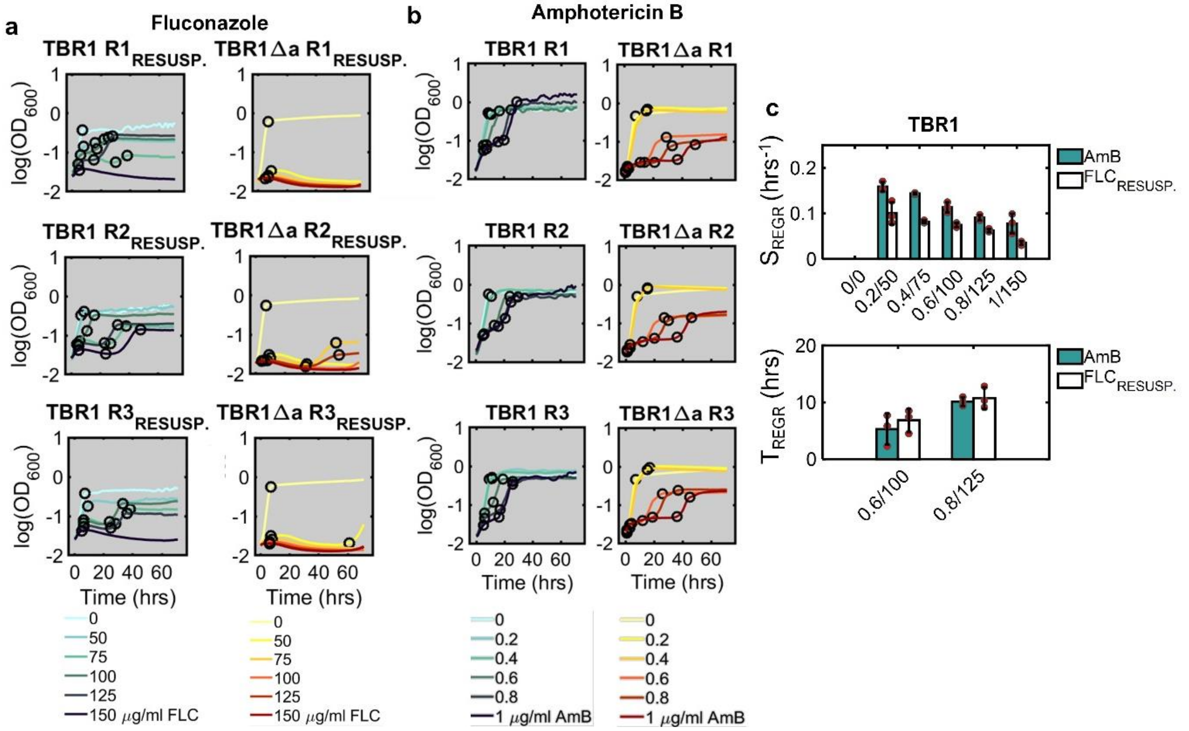

Supplementary Figure 14. Similarities between AmB and FLC growth response. a) TBR1 and TBR1Δa growth kinetics upon various FLC concentrations shown as log(OD_{600}) over 72 hours after resuspension. Black circles indicate the breakpoints defined by the piecewise linear fitting. **b)** Growth kinetics of TBR1 and TBR1Δa upon various AmB concentrations (duplicated from Figure 4B for visual comparison). **c)** Compared means of the pregrowth phase slopes (S_{PREGR}) and durations (T_{REGR}) upon AmB and FLC exposure. The x-axis shows the drug doses in [AmB]/[FLC] format. Red circles represent individual data points. Error bars represent means and standard deviations calculated from three biological replicates.

180

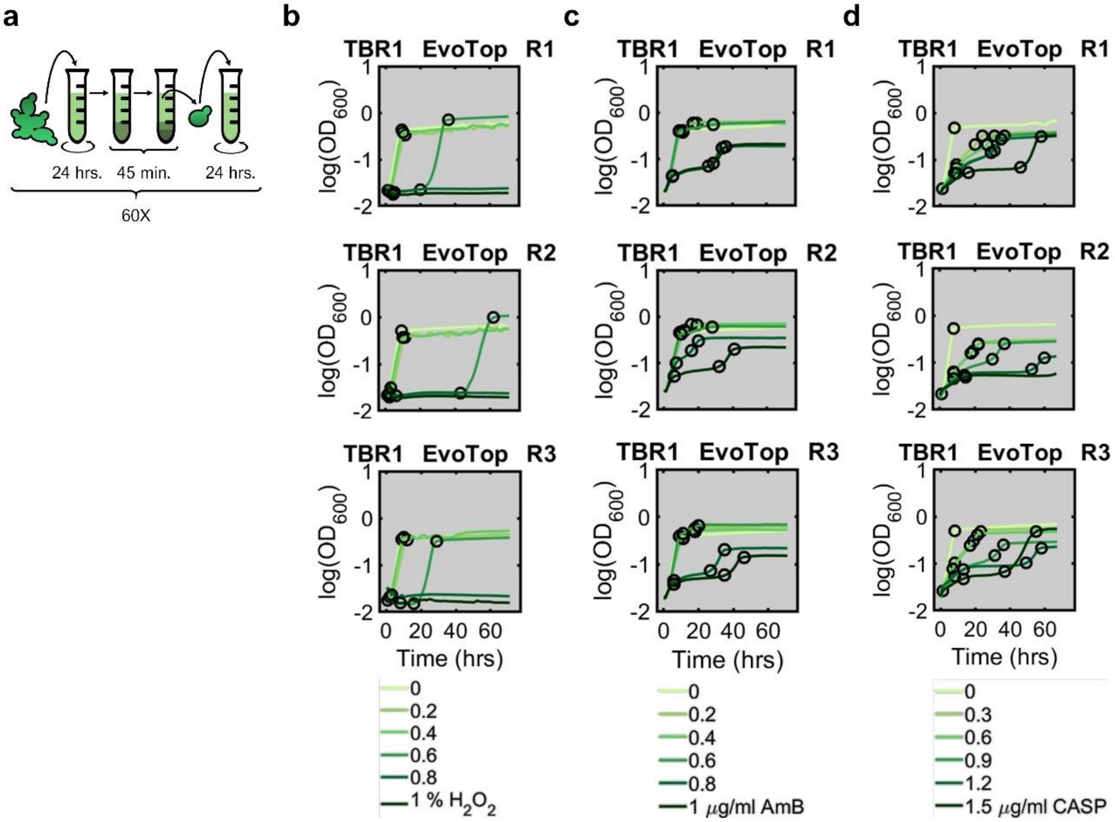

Supplementary Figure 15. TBR1 EvoTop strain growth response to H_2O_2 and AmB. a) Schematic illustration of the experimental evolution from clumping to unicellular TBR1 using gravitational selection[1]. Growth kinetics upon various concentrations of **b)** H_2O_2, **c)** AmB, and **d)** CASP shown as $\log(OD_{600})$ over 72 hours. Black circles indicate the breakpoints defined by the piecewise linear fitting.

181

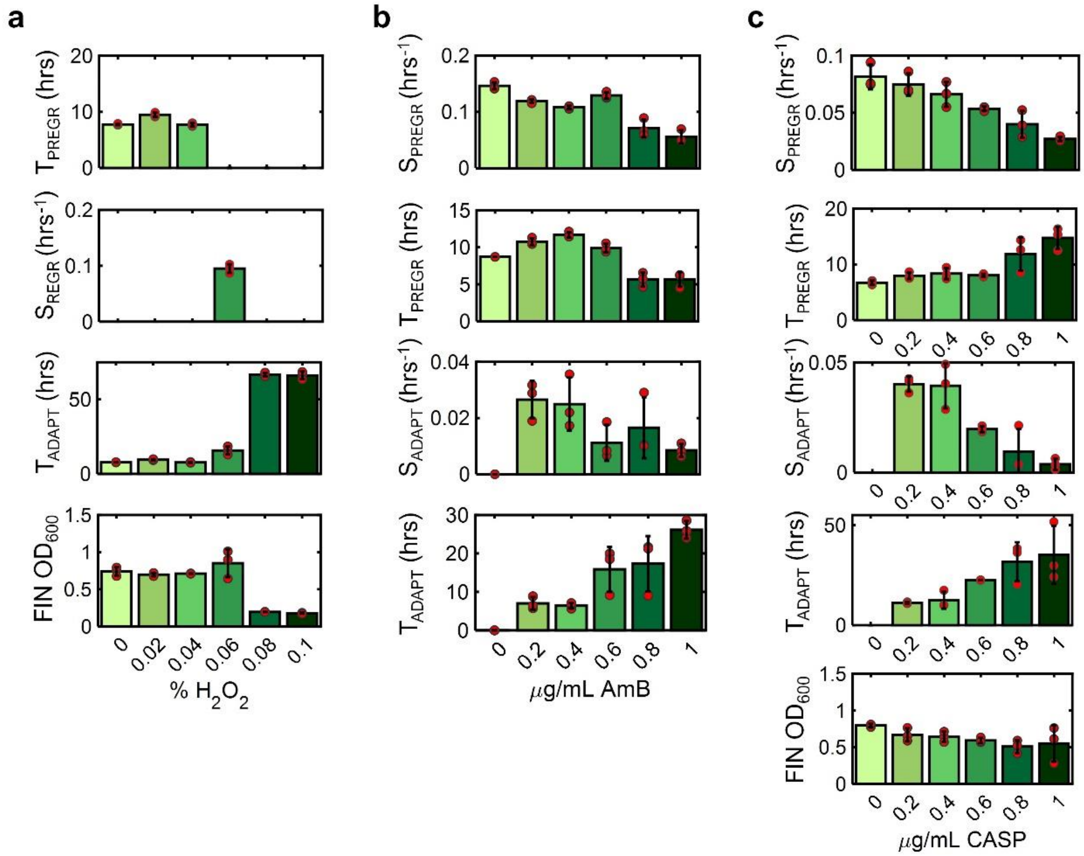

Supplementary Figure 16. Hydrogen peroxide (H_2O_2), amphotericin B (AmB), and caspofungin (CASP) effects on the most relevant growth phase parameters of TBR1 EvoTop. a) The duration of the pregrowth phase (T_{PREGR}), slope of the regrowth phase (S_{REGR}), duration of the adaptation phase (T_{ADAPT}), and the average final phase OD_{600} value (FIN OD_{600}) of TBR1 EvoTop strain in various H_2O_2 concentrations. **b)** The slope and duration of the pregrowth phase (S_{PREGR}, T_{PREGR}), slope and duration of the adaptation phase (S_{ADAPT}, T_{ADAPT}) of TBR1 EvoTop strain in various AmB concentrations. **c)** The slope and duration of the pregrowth phase (S_{PREGR}, T_{PREGR}), slope and duration of the adaptation phase (S_{ADAPT}, T_{ADAPT}), and the average final phase OD_{600} value (FIN OD_{600}) of TBR1 EvoTop strain in various CASP concentrations. Red circles represent individual data points. Error bars represent means and standard deviations calculated from three biological replicates.

a

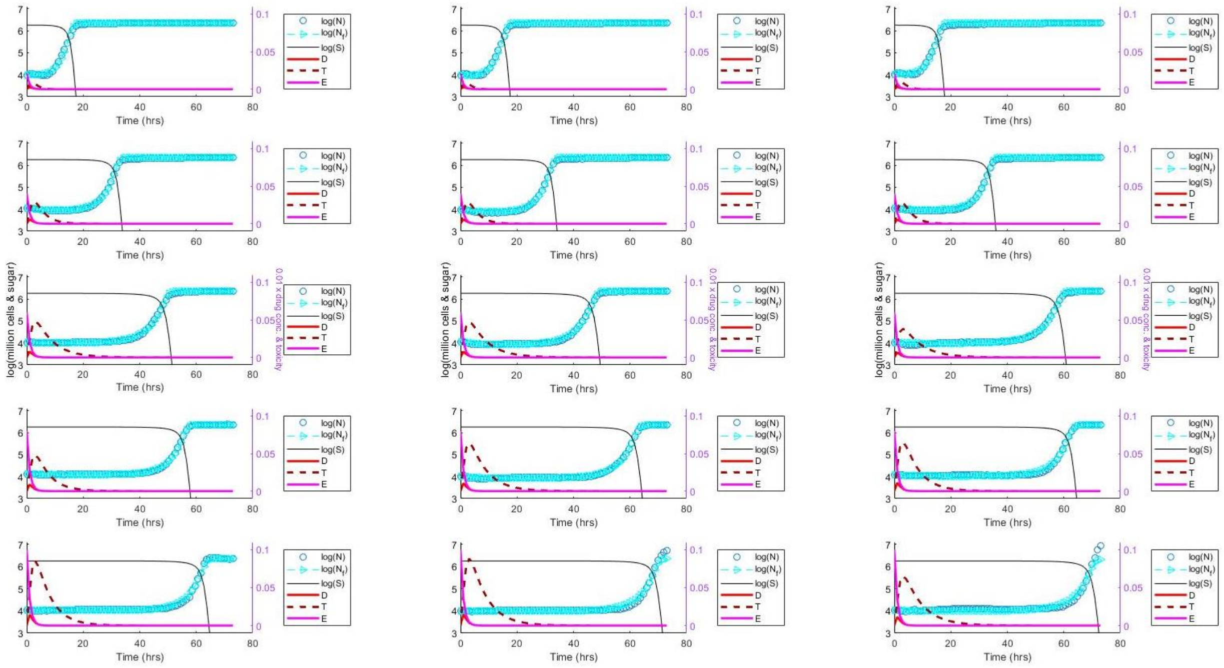

b

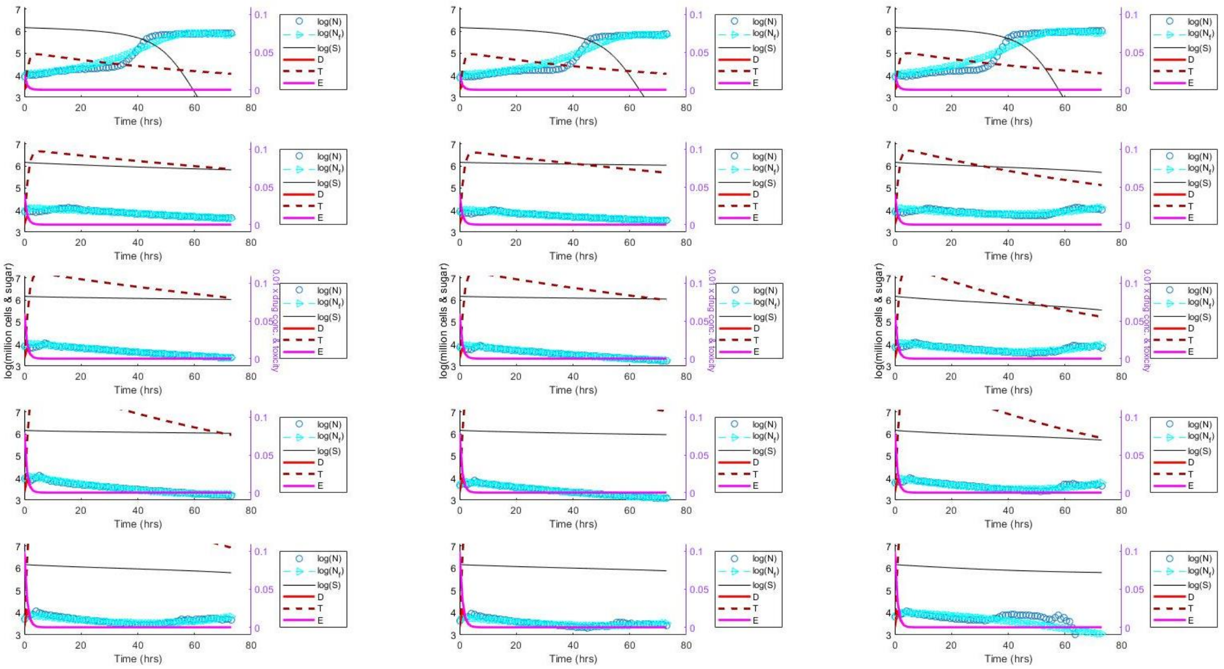

Supplementary Figure 17. H_2O_2 effects model parameter dynamic. Simulated growth curves (cyan triangles) produced by the model shown in **Figure 6a** plotted over the experimental cell count-converted growth curve data (blue circles) for **a)** TBR1 and **b)** TBR1Δa in three replicates (column 1, 2, 3, respectively) at five stress levels (0.02, 0.04, 0.06, 0.08, and 0.1% H_2O_2, rows 1-5, respectively). Simulated sugar (S), external drug (E), cellular toxicity (T), and internal drug (D) concentrations are plotted alongside.

a

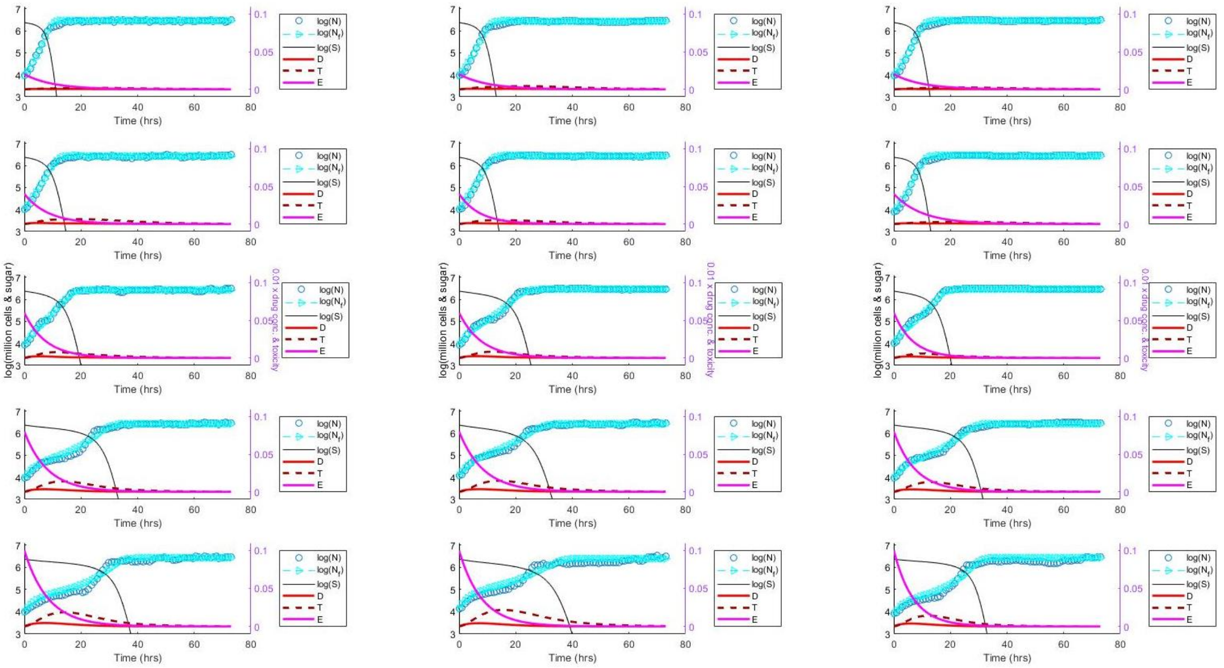

b

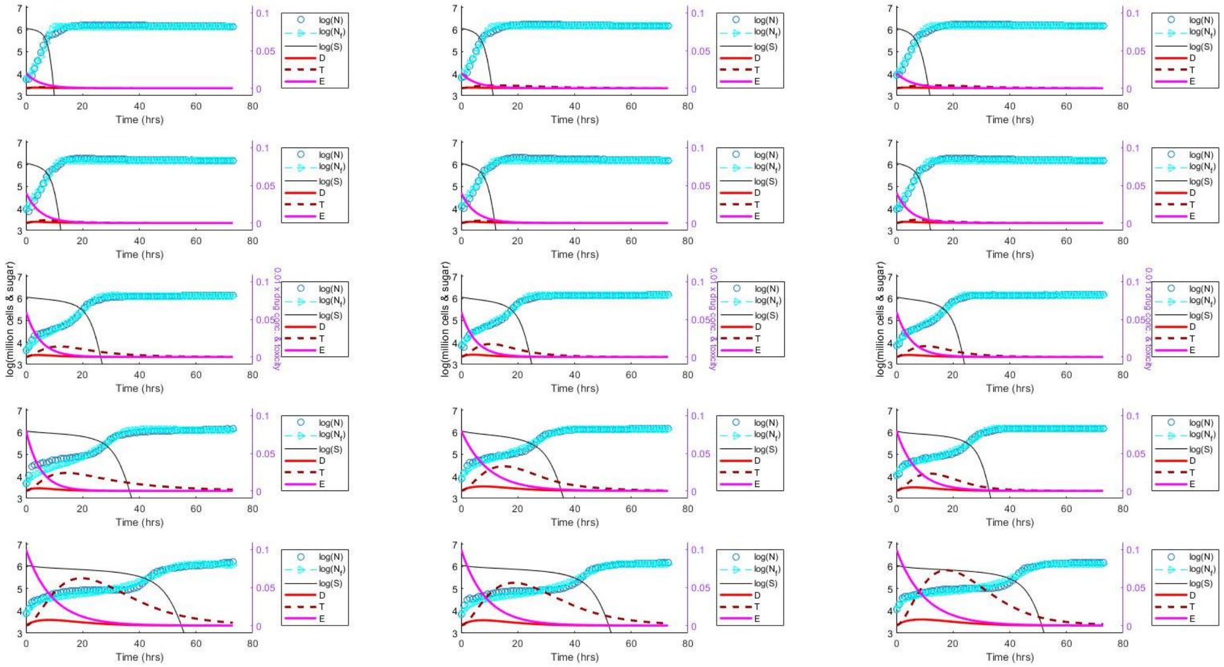

Supplementary Figure 18. AmB effects model parameter dynamic. Simulated growth curves (cyan triangles) produced by the model shown in **Figure 6a** plotted over the experimental cell count-converted growth curve data (blue circles) for **a)** TBR1 and **b)** TBR1Δa in three replicates (column 1, 2, 3, respectively) at five stress levels (0.2, 0.4, 0.6, 0.8, and 1 µg/mL AmB, row 1-5, respectively). Simulated sugar (S), external drug (E), cellular toxicity (T), and internal drug (D) concentrations are plotted alongside.

a

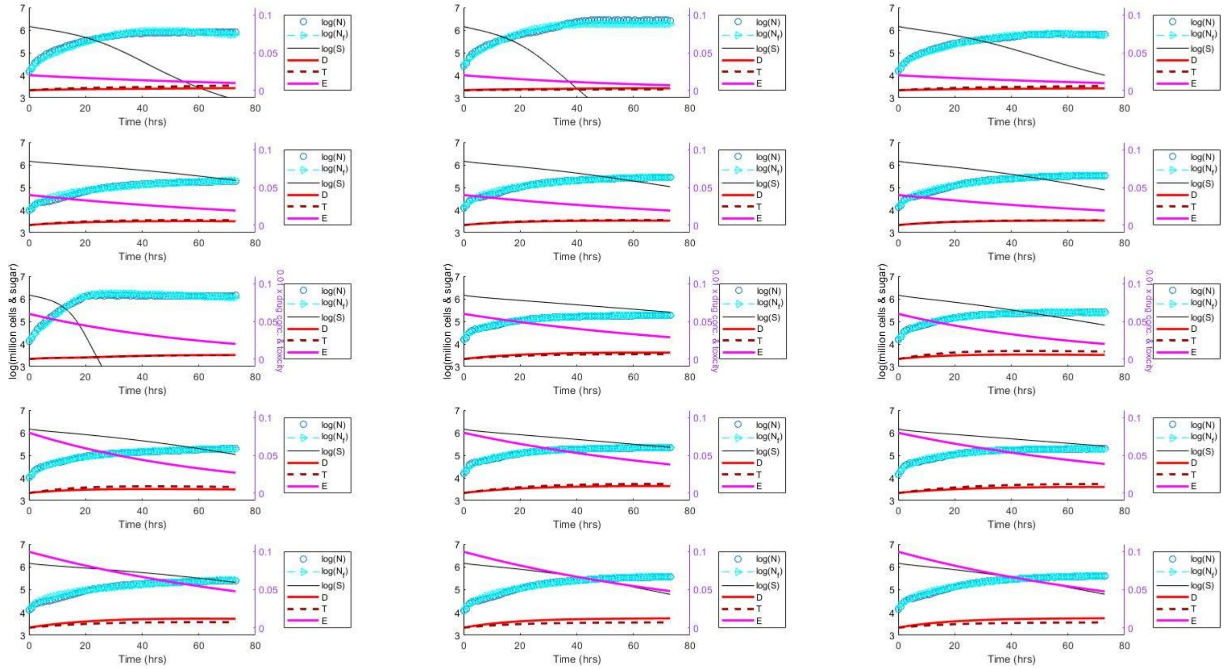

b

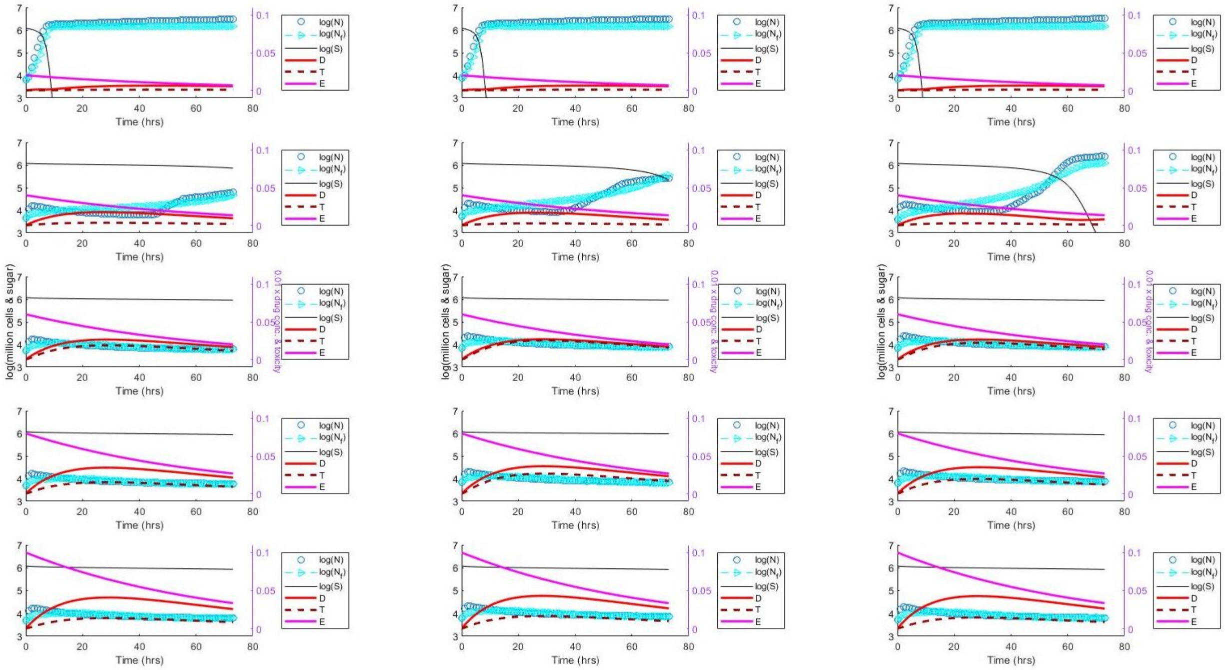

Supplementary Figure 19. CASP effects model parameter dynamic. Simulated growth curves (cyan triangles) produced by the model shown in **Figure 6a** plotted over the experimental cell count-converted growth curve data (blue circles) for **a)** TBR1 and **b)** TBR1Δa in three replicates (column 1, 2, 3, respectively) at five stress levels (0.2, 0.4, 0.6, 0.8, and 1 μg/mL CASP, row 1-5, respectively). Simulated sugar (S), external drug (E), cellular toxicity (T), and internal drug (D) concentrations are plotted alongside.

a

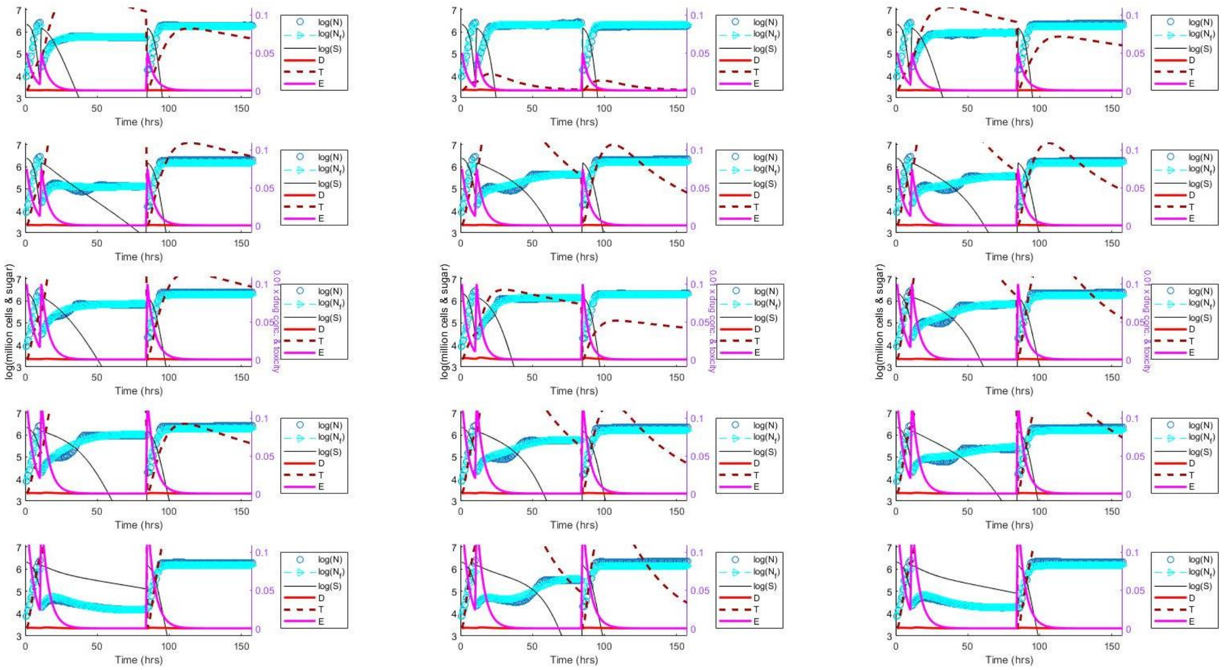

b

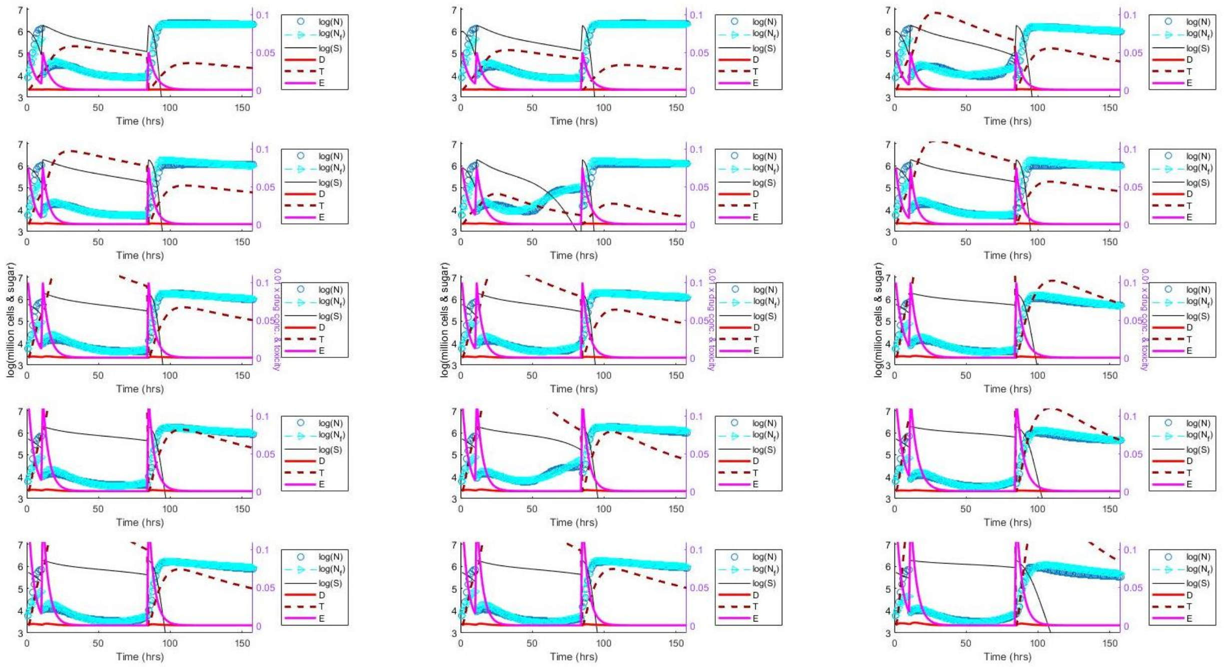

Supplementary Figure 20. FLC effects model parameter dynamic. Simulated growth curves (cyan triangles) produced by the model shown in **Figure 6a** plotted over the experimental cell count-converted growth curve data (blue circles) for **a)** TBR1 and **b)** TBR1Δa in three replicates (column 1, 2, 3, respectively) at five stress levels (50, 75, 100, 125, and 150 µg/mL FLC, row 1-5, respectively). Three time blocks in each plot represent pregrowth (10 hrs pre-resuspension), resuspension growth (~72 hrs post-resuspension), and growth with no resuspension (~72 hrs). Simulated sugar (S), external drug (E), cellular toxicity (T), and internal drug (D) concentrations are plotted alongside.

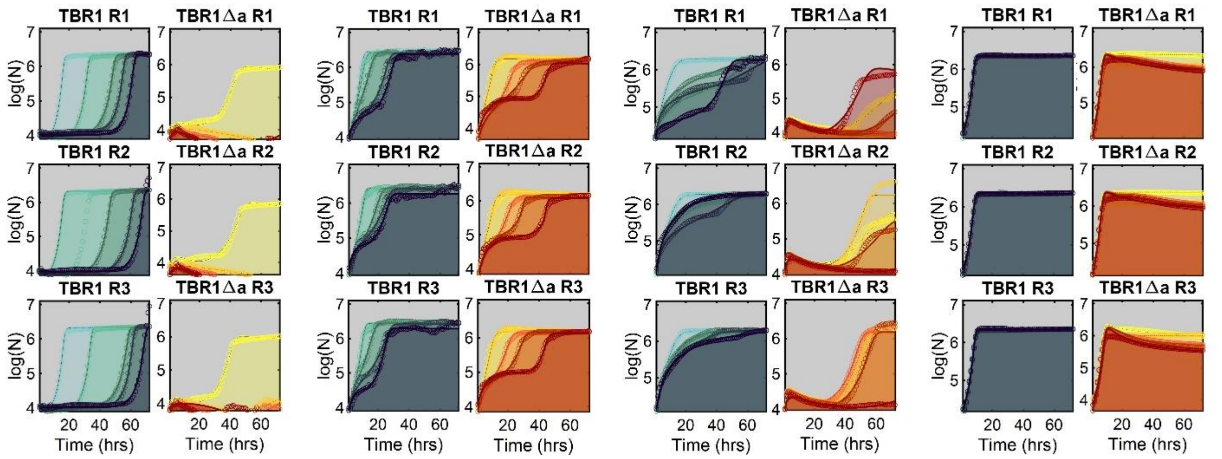

Supplementary Figure 21. Drug-affected AUC. ODE (solid lines) model of the experimental cell count-converted growth curves (dotted lines) and the area under the curve (AUC) of the model data shown as the shaded area for TBR1 (shades of blue) and TBR1Δa (shades of yellow). Color gradient from brighter to darker shades represents the increasing stress levels of H_2O_2 (0.02, 0.04, 0.06, 0.08, 0.1 %), AmB (0.2, 0.4, 0.6, 0.8, 1 µg/mL), CASP (0.2, 0.4, 0.6, 0.8, 1 µg/mL), and FLC (50, 75, 100, 125, 150 µg/mL).

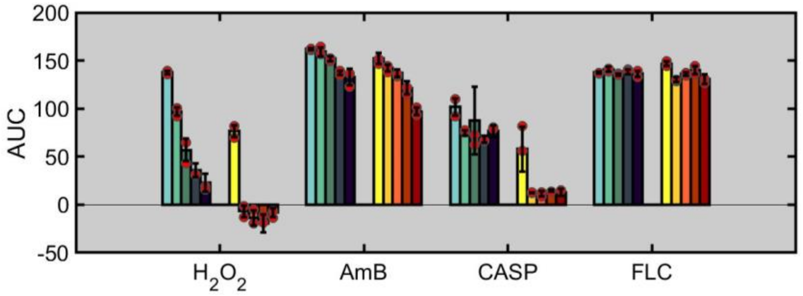

Supplementary Figure 22. The AUC of the simulated curves shown in Figure 6c (bars). Color gradient from brighter to darker shades represents the increasing stress levels of H_2O_2 (0.02, 0.04, 0.06, 0.08, 0.1 %), AmB (0.2, 0.4, 0.6, 0.8, 1 µg/mL), CASP (0.2, 0.4, 0.6, 0.8, 1 µg/mL), and FLC (50, 75, 100, 125, 150 µg/mL). Red circles represent individual AUC data points from replicate simulations. Error bars represent means and standard deviations calculated from the fits to three experimental replicates.

188

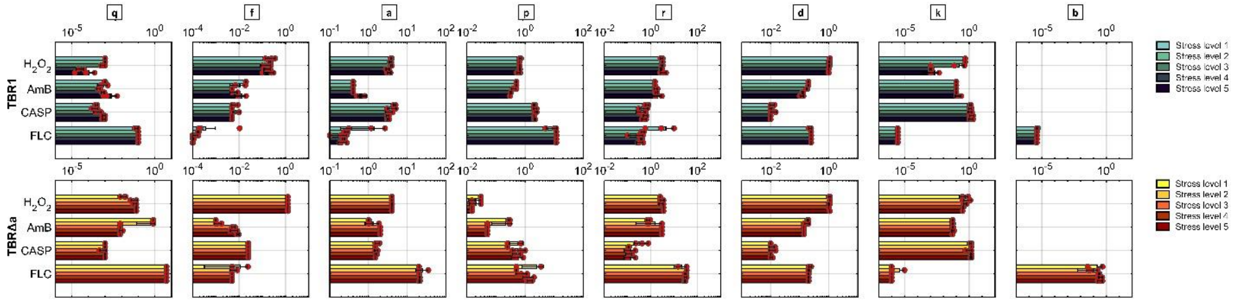

Supplementary Figure 23. The parameters for growth curve model of the cell count-converted growth curves (dotted lines) obtained upon intense shaking. Q – the drug threshold to inhibit the growth, f – drug influx rate, a – drug-induced cell toxicity, p – drug response production rate, r – the drug threshold to induce the response production, d – spontaneous drug decay, k – cell killing/death rate. Error bars represent means and standard deviations calculated from the ODE models of three experimental biological replicates. Color gradient from brighter to darker shades represents the increasing stress levels of H_2O_2, AmB, CASP, and FLC.

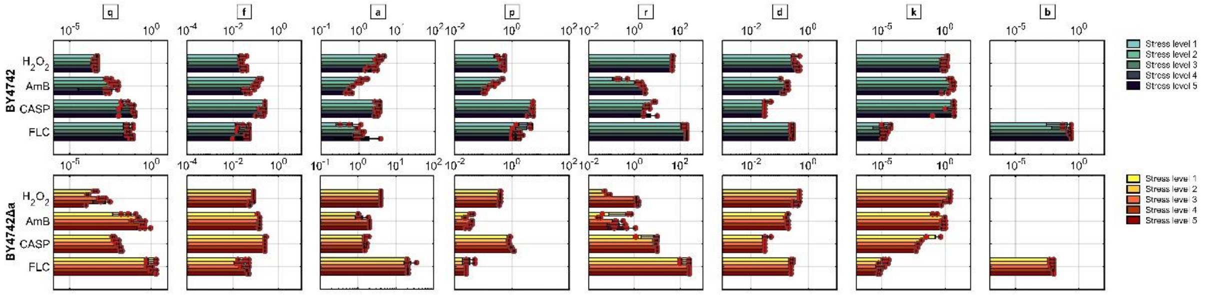

Supplementary Figure 24. The parameters for growth curve model of the cell count-converted growth curves (dotted lines) of BY4742 and BY4742Δa. Q – the drug threshold to inhibit the growth, f – drug influx rate, a – drug-induced cell toxicity, p – drug response production rate, r – the drug threshold to induce the response production, d – spontaneous drug decay, k – cell killing/death rate. Error bars represent means and standard deviations calculated from the ODE models of three experimental biological replicates. Color gradient from brighter to darker shades represents the increasing stress levels of H_2O_2, AmB, CASP, and FLC.

Supplementary References

1 Gandhi, S. R., Korolev, K. S. & Gore, J. Cooperation mitigates diversity loss in a spatially expanding microbial population. *Proc Natl Acad Sci U S A* **116**, 23582-23587, doi:10.1073/pnas.1910075116 (2019).

2 Kuzdzal-Fick, J. J., Chen, L. & Balázsi, G. Disadvantages and benefits of evolved unicellularity versus multicellularity in budding yeast. *Ecology and Evolution* **9**, 8509-8523, doi:10.1002/ece3.5322 (2019).
