# Supplementary material for: Drug-dependent growth curve reshaping reveals mechanisms of antifungal resistance in Saccharomyces cerevisiae
Source: Commun Biol. 2022 Mar 31;5:292. doi: 10.1038/s42003-022-03228-9 (PMC8971432; doi:10.1038/s42003-022-03228-9)
Supplement: Supplementary file 3 — Description of Additional Supplementary Files [file 42003_2022_3228_MOESM3_ESM.pdf]

Description of Additional Supplementary Files

File name: Supplementary Data 1
Description: p-value 488 calculations and relevant parameters.
