# Supplementary material for: Drug-dependent growth curve reshaping reveals mechanisms of antifungal resistance in Saccharomyces cerevisiae
Source: Commun Biol. 2022 Mar 31;5:292. doi: 10.1038/s42003-022-03228-9 (PMC8971432; doi:10.1038/s42003-022-03228-9)
Supplement: Supplementary file 5 — Reporting Summary [file 42003_2022_3228_MOESM5_ESM.pdf]

nature research

Corresponding author(s): Gábor Balázsi

Last updated by author(s): Jan 10, 2022

Reporting Summary

Nature Research wishes to improve the reproducibility of the work that we publish. This form provides structure for consistency and transparency in reporting. For further information on Nature Research policies, see our Editorial Policies and the Editorial Policy Checklist.

Statistics

For all statistical analyses, confirm that the following items are present in the figure legend, table legend, main text, or Methods section.

n/a	Confirmed	
☐	☒	The exact sample size (*n*) for each experimental group/condition, given as a discrete number and unit of measurement
☐	☒	A statement on whether measurements were taken from distinct samples or whether the same sample was measured repeatedly
☐	☒	The statistical test(s) used AND whether they are one- or two-sided<br>*Only common tests should be described solely by name; describe more complex techniques in the Methods section.*
☐	☒	A description of all covariates tested
☐	☒	A description of any assumptions or corrections, such as tests of normality and adjustment for multiple comparisons
☐	☒	A full description of the statistical parameters including central tendency (e.g. means) or other basic estimates (e.g. regression coefficient) AND variation (e.g. standard deviation) or associated estimates of uncertainty (e.g. confidence intervals)
☐	☒	For null hypothesis testing, the test statistic (e.g. *F*, *t*, *r*) with confidence intervals, effect sizes, degrees of freedom and *P* value noted<br>*Give P values as exact values whenever suitable.*
☒	☐	For Bayesian analysis, information on the choice of priors and Markov chain Monte Carlo settings
☒	☐	For hierarchical and complex designs, identification of the appropriate level for tests and full reporting of outcomes
☒	☐	Estimates of effect sizes (e.g. Cohen's *d*, Pearson's *r*), indicating how they were calculated

Our web collection on statistics for biologists contains articles on many of the points above.

Software and code

Policy information about availability of computer code

Data collection	Cellometer® Vision CBA Image Cytometer (Nexcelom Bioscience LLC.); Tecan Infinite 200Pro Microplate Reader; MATLAB R2020b
Data analysis	Tecan i-control (Version 2.0.10.0); Nexcelom Data Analysis Package; Microsoft® Excel® for Microsoft 365 MSO (Version 2111 Build 16.0.14701.20240) 32-bit; MATLAB R2020b

For manuscripts utilizing custom algorithms or software that are central to the research but not yet described in published literature, software must be made available to editors and reviewers. We strongly encourage code deposition in a community repository (e.g. GitHub). See the Nature Research guidelines for submitting code & software for further information.

Data

Policy information about availability of data

All manuscripts must include a data availability statement. This statement should provide the following information, where applicable:

- Accession codes, unique identifiers, or web links for publicly available datasets
- A list of figures that have associated raw data
- A description of any restrictions on data availability

The datasets are available at the Balázsi Lab Website at https://openwetware.org/wiki/CHIP:Data#Drug-Dependent_Growth_Curve_Reshaping_Reveals_Mechanisms_of_Antifungal_Resistance:_[14]

Field-specific reporting

Please select the one below that is the best fit for your research. If you are not sure, read the appropriate sections before making your selection.

☒ Life sciences ☐ Behavioural & social sciences ☐ Ecological, evolutionary & environmental sciences

For a reference copy of the document with all sections, see nature.com/documents/nr-reporting-summary-flat.pdf

Life sciences study design

All studies must disclose on these points even when the disclosure is negative.

Sample size	All analyses utilized complete experimental or simulated data sets. No sample-size calculation was performed. Sample size of the microscopy data was obtained from the Nexcelom Data Package image segmentation tool and indicated the number of (i) "single cells"; (ii) "clumps"; OR (iii) both "single cells" and "clumps"per sample volume. Sample size of the experimental (and modeled) growth curves was >=3 for the absorbance (OD600) at all time points, automatically acquired by the Tecan Infinite 200Pro via the i-control 2.0.10.0 software. Drug treatment experiments were performed in triplicates and analyzed independently.
Data exclusions	No data were excluded.
Replication	Growth curve data was obtained from three biological replicates (from three inoculates of genetically identical clones), analyzed independently with identical algorithms, fit, and simulated individually for each replicate in MATLAB R2020b. Microscopy measurements were repeated four times and a representative replicate was shown in the manuscript (the others can be found in the online data repository).
Randomization	The budding yeast strains were allocated into experimental groups based on genetic background (TBR1, TBR1Δa, BY4742, BY4742Δa, YPH500, KV38). Growth curves in drug-containing media were grouped by drug type and drug concentration. Curve parameters were grouped by slopes and durations of each growth phase as defined by the piecewise linear fits. The computational model output was categorized by the estimated parameters, using the values produced by the fits.
Blinding	Human subjects were not involved in this study. Due to the nature of these experiments, blinding was not applied.

Reporting for specific materials, systems and methods

We require information from authors about some types of materials, experimental systems and methods used in many studies. Here, indicate whether each material, system or method listed is relevant to your study. If you are not sure if a list item applies to your research, read the appropriate section before selecting a response.

Materials & experimental systems

n/a	Involved in the study	
☒	☐	Antibodies
☐	☒	Eukaryotic cell lines
☒	☐	Palaeontology and archaeology
☒	☐	Animals and other organisms
☒	☐	Human research participants
☒	☐	Clinical data
☒	☐	Dual use research of concern

Methods

n/a	Involved in the study	
☒	☐	ChIP-seq
☒	☐	Flow cytometry
☒	☐	MRI-based neuroimaging

Eukaryotic cell lines

Policy information about cell lines

Cell line source(s)	Todd B. Reynolds lab (TBR1), Yeast KO Collection (BY4742, BY4742Δa), James J. Collins lab (YPH500), Kevin Vestrepen lab (KV38), created in this lab (TBR1 EvoTop, TBR1Δa)
Authentication	We authenticated the cell lines by phenotypic observation: clumping, selection agents.
Mycoplasma contamination	Not relevant (no mammalian cell lines).
Commonly misidentified lines (See ICLAC register)	Not applicable.
